# PAI-1: A Key Signal at the Crossroads of Stem Cell Differentiation and Senescence

**DOI:** 10.3390/ijms27010086

**Published:** 2025-12-21

**Authors:** Jihan Ke, Youping Jiang, Zhiyong Cheng, Yulan Zhou, Jiaxu Lu, Bo Xu, Shouquan Yan, Jiafeng Wang

**Affiliations:** 1 Stem Cell Research and Cellular Therapy Center, Affiliated Hospital of Guangdong Medical University, Zhanjiang 524001, China; kejihan@gdmu.edu.cn (J.K.); jumpingjyp@163.com (Y.J.); czylgd666@163.com (Z.C.); ljiaxu@126.com (J.L.); xb13113086762021@163.com (B.X.); wdyx866@163.com (S.Y.); 2 Reproductive Medicine Center, Affiliated Hospital of Guangdong Medical University, Zhanjiang 524001, China; zyl092078@163.com

**Keywords:** PAI-1, differentiation, aging, senescence, degenerative diseases

## Abstract

Plasminogen activator inhibitor-1 (PAI-1) is a central regulator of the fibrinolytic system and is increasingly recognized for its pivotal roles in a broad spectrum of physiological and pathological processes. In addition to its classical function in fibrinolysis, accumulating evidence highlights the involvement of PAI-1 in cellular senescence, differentiation, fibrosis, thrombosis, and tumorigenesis. This review systematically summarizes recent advances in understanding the multifaceted biological functions of PAI-1, with a particular emphasis on its dual regulatory roles in cellular differentiation and senescence. Through manual curation and analysis of the literature, we constructed a PAI-1-centered signaling network associated with differentiation and further integrated this framework with known senescence-related pathways. This integrative approach aims to elucidate the crosstalk between differentiation and senescence mediated by PAI-1. By providing an in-depth overview of PAI-1 functions across various experimental models, this review offers a theoretical foundation for exploring its potential as a therapeutic target and presents novel perspectives for the development of intervention strategies for complex chronic diseases.

## 1. Introduction

Plasminogen activator inhibitor-1 (PAI-1), a crucial member of the serine protease inhibitor (serpin) superfamily, is encoded by the *SERPINE1* gene located on chromosome 7q21.3-q22 in humans. The gene spans approximately 12.3 kb and consists of 9 exons and 8 introns [1]. The translated product is a glycoprotein comprising 379/381 amino acids with a molecular weight of approximately 47 kDa.PAI-1 lacks disulfide bonds and exists in an intrinsically unstable active conformation, with a physiological half-life of only 1–2 h [2,3]. PAI-1 was initially discovered in the culture medium of bovine aortic endothelial cells by D. J. Loskutoff and colleagues in 1983 [4]. In 1986, four independent research groups successfully cloned human PAI-1 cDNA and confirmed its identity as the principal physiological inhibitor of tissue-type and urokinase-type plasminogen activators (tPA/uPA) [5]. In the 1990s, studies using *PAI-1* knockout mice further validated its essential antifibrinolytic function while also revealing a notable species difference—fibrinolysis in mice was found to be approximately ten times slower than that in humans [6]. In recent decades, research has revealed the pleiotropic functions of PAI-1, including its involvement in regulating cell migration, angiogenesis, and senescence, establishing it as a hallmark of cellular senescence [7,8].

Endothelial cells represent the primary source of PAI-1 production, and its expression can be markedly upregulated by inflammatory cytokines such as TNF-α, IL-1β, and various metabolic stimuli [9]. In addition to being expressed in endothelial cells, PAI-1 is also expressed in smooth muscle cells, hepatocytes, adipocytes, megakaryocytes, and platelets. In platelets, it is primarily stored in α-granules and circulates in the plasma [10,11]. Upon platelet activation, PAI-1 is released and can remain bound to the surface of activated platelets, contributing significantly to thrombus stabilization [12]. Platelets are considered the largest physiological reservoir of PAI-1, with plasma concentrations ranging from 200 to 300 ng/mL; however, only approximately 10% of circulating PAI-1 retains biological activity [6]. Some studies have reported the presence of *PAI-1* mRNA in anucleated cells, suggesting the capacity for de novo synthesis [13]. PAI-1 is widely expressed across multiple tissues, particularly in the cardiovascular system and adipose tissue [14]. Under physiological conditions, its plasma concentration typically ranges between 5 and 20 ng/mL [15].

Initially, recognized for its pivotal role in hemostasis through the regulation of fibrinolysis, PAI-1 exerts its antifibrinolytic effects by inhibiting tPA and uPA, thereby suppressing the conversion of plasminogen to plasmin. At sites of vascular injury, PAI-1 cooperates with activated platelets to block plasmin formation and thus stabilize thrombi [16].

**Figure 1:**
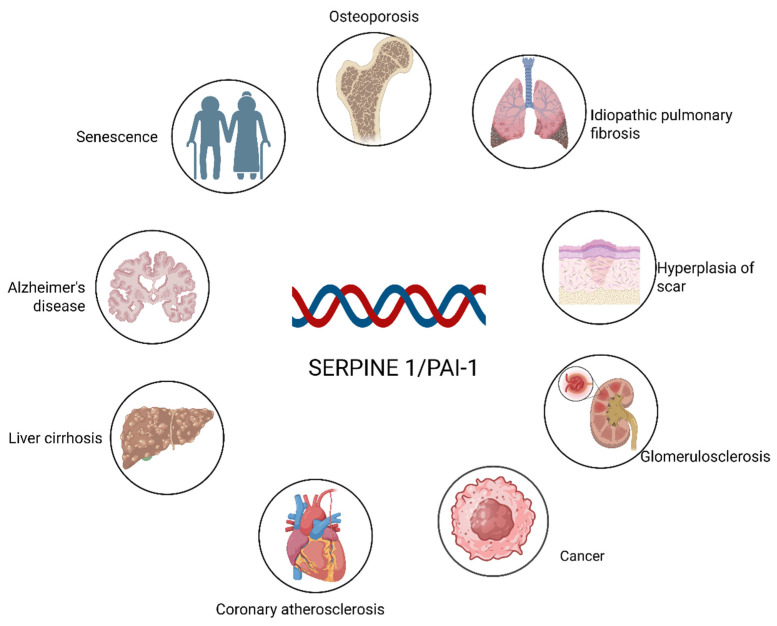
SERPINE1/PAI-1 as a key driver in multiorgan pathologies. The figure depicts the widespread impact of PAI-1 across various physiological systems. Beyond its role in fibrinolysis, PAI-1 contributes to the pathogenesis of major fibrosis-related diseases (lung, liver, kidney, and skin), neurodegeneration (Alzheimer’s), and cardiovascular complications, while also serving as a hallmark of cellular senescence and aging-associated disorders. Created in BioRender. Ke, J. (2025) https://BioRender.com/dg7paf2.

Over the past several decades, accumulating evidence has demonstrated that PAI-1 functions not only as a central regulator of the fibrinolytic system but also as a key participant in a wide spectrum of pathophysiological processes (Figure 1). As illustrated, aberrant PAI-1 expression is implicated in neurodegenerative disorders such as Alzheimer’s disease [17] and cardiovascular conditions including coronary atherosclerosis [18]. Furthermore, PAI-1 acts as a pivotal driver of fibrosis across multiple organ systems, contributing to idiopathic pulmonary fibrosis, liver cirrhosis, glomerulosclerosis, and pathological scar hyperplasia [19,20]. Beyond these conditions, PAI-1 facilitates tumor progression in various cancers [21]. PAI-1 is a hallmark of cellular senescence and is closely linked to age-related phenotypes such as osteoporosis [22]. Among these diverse roles, its specific function in regulating the osteogenic differentiation of stem cells—a critical process for maintaining bone homeostasis—has drawn increasing attention. However, its regulatory effects appear to be influenced by factors such as the tissue microenvironment, spatial‒temporal conditions, and sex differences [23]. Our previous studies revealed that the upregulation of PAI-1 significantly enhances the differentiation of mesenchymal stem cells (MSCs) into osteoblasts [24].Conversely, given that PAI-1 accelerates senescence [8], elucidating strategies to balance its antifibrinolytic activity with its impact on stem cell fate represents an important research focus.

In this review, we provide a comprehensive summary of the latest advances in PAI-1 biology, extending beyond its established function in hemostasis. We systematically examine its pathogenic roles in cardiovascular disease, metabolic syndromes such as diabetes, and multi-organ fibrosis—specifically highlighting pulmonary and renal pathologies. Furthermore, we explore its critical involvement in tumorigenesis, aging, and stem cell fate, with a particular focus on osteogenic differentiation. By integrating these diverse contexts, we aim to offer novel insights and theoretical foundations for future research in this evolving field.

### Methodology and Scope of Review

To ensure a rigorous synthesis of the current literature, we conducted a comprehensive search using databases including PubMed, Web of Science, and Google Scholar. The search strategy employed combinations of keywords such as “PAI-1/*SERPINE1*,” “cellular senescence,” “differentiation,” “osteogenesis,” “fibrosis,” and “Bone metabolic diseases.” We prioritized peer-reviewed original research and systematic reviews published within the last decade, while also retaining seminal foundational studies to provide historical context. Inclusion criteria focused on studies clarifying the molecular mechanisms of PAI-1 in age-related diseases and stem cell fate determination. It should be noted that the “PAI-1-centered signaling network” and schematic illustrations presented herein represent a manually curated conceptual synthesis derived from the integration of experimental evidence across diverse tissue contexts, rather than any bioinformatic or systems level analysis. This approach allows for the construction of an integrative framework connecting PAI-1 to pleiotropic cell fate decisions.

## 2. Physiological Roles of PAI-1

Before discussing the deleterious effects of PAI-1 in aging and disease, it is crucial to recognize its fundamental role in maintaining physiological homeostasis. Under healthy conditions, PAI-1 expression is kept at low basal levels but is rapidly inducible in response to injury or stress, serving as a protective “molecular brake.”

The primary physiological function of PAI-1 is to prevent excessive bleeding at sites of vascular injury. By inhibiting tPA and uPA, PAI-1 stabilizes the nascent fibrin clot, providing a temporary scaffold for tissue repair. Beyond hemostasis, PAI-1 regulates cell migration during wound healing. By interacting with the uPA/uPAR complex and vitronectin, PAI-1 controls the detachment of cells from the extracellular matrix (ECM), a process essential for keratinocyte migration and re-epithelialization [18].

In response to acute stress or DNA damage, a transient upregulation of PAI-1 induces a temporary state of cellular senescence. This is physiologically beneficial as it halts the proliferation of damaged cells and limits fibrosis by checking excessive ECM degradation during the early phase of repair [25]. However, when PAI-1 expression becomes chronic—due to unresolved inflammation, metabolic dysfunction, or continuous aging signals—it becomes pathological. Sustained high levels of PAI-1 prevent the clearance of senescent cells, perpetuate the Senescence-Associated Secretory Phenotype (SASP), and lead to permanent tissue remodeling and organ failure [26].

## 3. *SERPINE1* Gene and Diseases

### 3.1. SERPINE1 Polymorphism and Diseases Risk

A guanine (G) insertion/deletion polymorphism at position −675 in the promoter region of the *PAI-1* gene results in two distinct alleles, designated 4G and 5G, which are collectively referred to as the PAI-1 4G/5G polymorphism. The 4G allele has a relatively high affinity for transcriptional activators, leading to increased mRNA transcription and increased PAI-1 expression. Conversely, the 5G allele preferentially binds transcriptional repressors, thereby attenuating PAI-1 expression [27]. Individuals homozygous for the 4G allele (4G/4G genotype) typically present elevated plasma levels of PAI-1, reduced fibrinolytic capacity, and an increased risk of venous thrombosis [28]. In contrast, individuals with the 5G/5G genotype tend to achieve better venous thrombus recanalization and exhibit a lower risk of thrombosis recurrence [29].

Notably, the PAI-1 4G/5G polymorphism has been investigated for its link to susceptibility to type 2 diabetes mellitus (T2DM), though results remain a subject of debate. While T2DM is characteristically associated with elevated plasma PAI-1 levels, genetic studies have yielded conflicting findings: some meta-analyses suggest the 4G allele (associated with high expression) is a risk factor, whereas other observations, such as those in specific populations, have reported a higher frequency of the 5G allele [30]. This apparent inconsistency highlights that the relationship between *SERPINE1* genotype and diabetic risk is not a simple linear causality. Instead, the final pathological phenotype is likely shaped by a complex interplay of metabolic regulators (such as obesity and insulin resistance), environmental factors, and gene–gene interactions, which may override or modulate the direct effects of the promoter polymorphism itself. Furthermore, research investigating the association between *PAI-1* gene polymorphisms and osteoporotic vertebral compression fracture (OVCF) in postmenopausal women has proposed that such polymorphisms may aid in assessing OVCF risk and serve as potential genetic biomarkers [31].

Interestingly, a meta-analysis of patients with rheumatoid arthritis (RA) revealed no significant elevation in PAI-1 levels among RA patients or any definitive correlation between disease risk and the 4G/5G polymorphism [32]. Moreover, a study conducted by Daniela Mari et al. on centenarians revealed that the *PAI-1* −675 (4G/5G) polymorphism, including the prothrombotic 4G allele, as well as other coagulation-related mutations, such as factor V Leiden (Arg506Gln) and prothrombin G20210A, was more prevalent in long-lived individuals than in the general elderly population. These findings suggest that the presence of a procoagulant profile does not necessarily predispose patients to thrombosis and may, in fact, be compatible with healthy aging and longevity [33]. Therefore, the relationship between the *PAI-1* 4G/5G polymorphism and longevity remains inconclusive and may not be directly causal [34].

### 3.2. SERPINE1 Gene and Age-Related Diseases

(Table 1) Summarizes representative studies highlighting the critical role of PAI-1 in a wide range of diseases. The functions of PAI-1 are multifaceted, contributing to diverse physiological and pathological processes. The spectrum of disorders associated with PAI-1 dysregulation is broad and heterogeneous. Accumulating evidence has firmly established that PAI-1 serves as a key regulatory molecule in the development and progression of complex chronic diseases arising from both natural aging and pathological accelerated aging [26].

#### 3.2.1. Cardiovascular Disease and Diabetes

In the context of cardiovascular health, PAI-1 acts as more than just a fibrinolytic inhibitor; it is a critical mediator of vascular remodeling and endothelial dysfunction. As a pivotal biomarker, PAI-1 contributes substantially to the elevated risk of cardiovascular events by promoting a hypofibrinolytic state and facilitating pathological thrombus formation [35].Clinically, epidemiological evidence from the Framingham Heart Study and other cohorts has established that elevated plasma PAI-1 levels are strongly associated with and serve as an independent predictor of major adverse cardiovascular events (MACE) and myocardial infarction in humans [62]. While human studies primarily demonstrate correlation, direct experimental evidence from mouse models confirms a causal role for PAI-1 in vascular pathology. Mechanistically, elevated PAI-1 levels in the vessel wall promote neointimal hyperplasia by regulating the migration and proliferation of vascular smooth muscle cells (VSMCs) and inhibiting the degradation of ECM, a process essential for plaque stability and vascular integrity [2,63]. Furthermore, PAI-1 contributes to endothelial senescence via the limitation of Klotho expression and the induction of oxidative stress, thereby accelerating the progression of atherosclerosis [64].

In the pathogenesis of diabetes, PAI-1 serves as a molecular link between metabolic dysregulation and vascular complications. Elevated PAI-1 levels are directly driven by hyperglycemia, hyperinsulinemia, and elevated free fatty acids, which stimulate PAI-1 transcription via responsive elements in its promoter region [36]. This upregulation creates a pro-thrombotic environment. Moreover, in adipose tissue, PAI-1 expression is markedly increased in obesity and type 2 diabetes, where it interferes with insulin signaling pathways, thereby exacerbating insulin resistance. This vicious cycle not only heightens the thrombotic risk but also accelerates distinct diabetic vascular complications through sustained vascular inflammation [65].

#### 3.2.2. Pulmonary Fibrosis

The involvement of PAI-1 in IPF extends beyond fibrin deposition to the intricate regulation of alveolar epithelial cell (AEC) fate and SASP. In an early study, Rui-Ming Liu et al. demonstrated that PAI-1 induces senescence in alveolar type II epithelial cells (ATIIs) through activation of the p53-p21-pRB signaling pathway. Building on this, subsequent investigations revealed a proteolytic-independent mechanism wherein PAI-1 directly interacts with the proteasome to inhibit its activity. This inhibition stabilizes p53, preventing its degradation and enhancing downstream p53 signaling, which ultimately locks cells in a senescent state [37,38,39,40].

Crucially, this process creates a pro-fibrotic feedback loop. Senescent ATIIs secrete SASP factors, including TGF-β and IL-6, which further upregulate PAI-1 and stimulate nearby fibroblasts to differentiate into collagen-producing myofibroblasts. Since PAI-1 inhibits plasmin-mediated ECM degradation, the excessive collagen deposited by these myofibroblasts is not cleared effectively, leading to progressive lung scarring. Notably, the expression of PAI-1 is itself regulated by the p53/p21 axis, forming a complex autoregulatory feedback loop that perpetuates fibrosis progression [40].

#### 3.2.3. Renal Diseases and Neurodegenerative Disorders

In renal pathology, PAI-1 is a key driver of glomerulosclerosis and tubulointerstitial fibrosis. Fabiola Terzi et al. demonstrated in aging glomerular endothelial cells that elevated PAI-1 levels induce internalization of the uPAR/β1 integrin complex. This molecular event disrupts the podocyte cytoskeletal architecture, leading to podocyte detachment and apoptosis, which are early hallmarks of glomerulosclerosis [41]. Furthermore, mechanistic studies by Cody C. Gifford et al. reported that PAI-1-mediated depletion of the anti-aging protein Klotho alleviates the inhibition of p53 and TGF-β1 signaling. This loss of Klotho results in uncontrolled TGF-β1/SMAD3 signaling, driving renal tubular epithelial cells towards cell cycle arrest and a pro-fibrotic phenotype. The consequent increase in fibrotic factor secretion and ECM deposition ultimately leads to renal failure [66].

Regarding neurodegenerative diseases, PAI-1 plays a detrimental role in the central nervous system by compromising the blood–brain barrier (BBB) and impairing neurovascular coupling. PAI-1 has been implicated in the pathogenesis of multiple sclerosis and Alzheimer’s disease (AD). In AD, the PAI-1-mediated inhibition of tPA reduces the proteolytic clearance of amyloid-beta (Aβ) plaques, promoting their accumulation in the brain. Additionally, PAI-1 contributes to brain cell senescence and mediates neuronal apoptosis by promoting neuroinflammation and microglial activation. The regulation of PAI-1 expression thus plays a pivotal role in modulating neurotoxicity and neurodegenerative alterations associated with cognitive decline [67,68].

#### 3.2.4. Bone Metabolic Diseases

Alkebaier Aobulikasimu et al. reported that the SIRT6–PAI-1 axis plays a critical regulatory role in the aging process. Modulation of SIRT6 expression ameliorates age-related bone metabolic abnormalities by suppressing PAI-1 expression. As a key driver of aging, PAI-1 directly promotes osteocyte senescence and the release of SASP factors through activation of the p53-p21 signaling pathway [42]. Moreover, PAI-1, a component of the SASP, has been implicated in the senescence of chondrocytes. Arsenic trioxide (As_2_O_3_) significantly increases senescence-associated β-galactosidase (SA-β-Gal) activity and the expression of senescence-related proteins in human chondrocytes by activating the p38/JNK–p16/p21/p53 and GATA4–NF-κB–SASP signaling pathways. This activation leads to the secretion of SASP factors such as PAI-1, MMP13, and IL-1β, thereby accelerating cell cycle arrest and senescence progression [43]. In addition, Jianping Bi et al. reported that vilagliptin significantly inhibits TNF-α-induced p53-K382 acetylation in chondrocytes by activating the AMPK/SIRT1 signaling pathway. This inhibition reduces SA-β-Gal activity and the expression of p53, p21, and PAI-1, thereby preventing G1-phase cell cycle arrest and exerting anti-senescent effects in chondrocytes [69].

#### 3.2.5. Cancer

On the basis of analyses of extensive clinical databases, numerous studies have demonstrated that PAI-1 is markedly overexpressed in biopsy tissues or plasma from various tumor types compared with normal controls [16,44,45]. However, after years of comprehensive investigation, it has become evident that PAI-1 plays a dual role in tumorigenesis and progression, acting not only as a protumorigenic factor but also as a tumor suppressor in certain contexts. For example, in pancreatic cancer, PAI-1 has been shown to exert a pronounced tumor suppressor effect [16]. Conversely, in multiple solid tumors, including breast, gastric, and ovarian cancers, elevated PAI-1 expression within tumor tissues is frequently correlated with poor clinical prognosis [46], and circulating PAI-1 levels are regarded as potential prognostic biomarkers in patients with malignancies such as breast cancer [70].

As the principal inhibitor of plasminogen activator, PAI-1 was initially considered to impede tumor cell migration and metastasis by inhibiting fibrinolysis and restricting extracellular matrix degradation. Indeed, in certain cancers, such as pancreatic cancer, glioma, and melanoma, PAI-1 overexpression has been reported to suppress tumor cell migration and invasion [47,48]. Paradoxically, elevated PAI-1 expression is associated with increased tumor invasiveness and metastatic potential in malignancies such as osteosarcoma, head and neck squamous cell carcinoma, and breast cancer [49,71].

Furthermore, PAI-1 plays a pivotal role in regulating tumor angiogenesis, exhibiting concentration-dependent proangiogenic activity in vivo: physiological levels of PAI-1 promote angiogenesis, whereas supraphysiological expression of PAI-1 exerts inhibitory effects [72]. This biphasic behavior implies that PAI-1 may indirectly facilitate tumor dissemination and distant metastasis by modulating angiogenesis during tumor progression. Importantly, various stromal cells within the tumor microenvironment (TME) also secrete PAI-1, amplifying its tumor-promoting functions through complex intercellular crosstalk with tumor cells [45]. These findings underscore the critical role of PAI-1 as a key mediator of TME–tumor interactions.

## 4. *SERPINE1* Gene and Aging

Senescence is a complex biological process characterized by the progressive loss of proliferative capacity and the transition to a stable state of cell cycle arrest following a finite number of cell divisions or exposure to external stressors [73]. Although senescent cells cease to divide, they remain metabolically active and influence the surrounding microenvironment through the secretion of the SASP. The association between PAI-1 and cellular senescence was first identified by its elevated expression in senescent cells.

In 1991, Murano et al. reported that dermal fibroblasts isolated from patients with Werner syndrome exhibited premature senescence accompanied by a marked increase in PAI-1 expression. Werner syndrome is clinically characterized by early-onset alopecia, diabetes, and osteoporosis [50]. In 1994, Goldstein et al. reported significantly lower PAI-1 expression in fibroblasts derived from fetal and neonatal mice than in those derived from aged mice; similarly, early-passage cultured fibroblasts presented lower PAI-1 levels than late-passage cells did [74]. In 1995, Comi et al. demonstrated significant upregulation of *PAI-1* mRNA and protein expression in senescent endothelial cells, whereas such changes were absent in early-passage cells, which were arrested because of contact inhibition [51].

In 2000, Xu et al. reported that prolonged exposure of endothelial cells to homocysteine (Hcy) induced increased expression of intercellular adhesion molecule-1 (ICAM-1) and PAI-1, both of which are closely linked to endothelial senescence [52]. Tianjiao Sun et al. reported that PAI-1 modulates Hcy-induced vascular endothelial senescence via the integrin β3 and oxidative stress pathways, highlighting its potential as a therapeutic target for cardiovascular diseases [53]. In 2010, McDonald et al. reported significantly elevated plasma PAI-1 levels in aged mice with thrombosis compared with young or age-matched nonthrombosed controls, implicating PAI-1 as a key mediator of vascular lesions and age-related thrombosis driven by endothelial senescence [54].

With increasing passage number, cells progressively acquire senescent phenotypes characterized by the upregulation of PAI-1 and various cell cycle regulators. Compared with their low-passage counterparts, high-passage (p30) human umbilical vein endothelial cells (HUVECs) exhibit significantly reduced proliferation during in vitro culture [55]. Moreover, Sirt1 inhibition enhances p53 acetylation and induces premature senescence-like phenotypes accompanied by increased PAI-1 and decreased endothelial nitric oxide synthase (eNOS) expression, suggesting a protective role of Sirt1 in endothelial cells via this pathway [56]. Another study showed that streptozotocin-induced chronic hyperglycemia promotes vascular senescence in murine aortas, as evidenced by elevated PAI-1, p53, and p21 expression; SIRT1 overexpression ameliorated these effects and prevented the downregulation of manganese superoxide dismutase (MnSOD) induced by hyperglycemia [57]. An additional critical mechanism by which PAI-1 induces senescence involves its interaction with insulin-like growth factor binding protein 3 (IGFBP3). Elzi et al. identified IGFBP3 as a key SASP component that induces cellular senescence in breast cancer cells post-chemotherapy, with its function modulated by tPA-mediated proteolysis. This finding suggests that IGFBP3 is a vital downstream effector in the PAI-1-mediated extracellular senescence regulatory cascade [58]. Zhang et al. further revealed that loss of the RNA-binding protein RBM4 leads to downregulation of miR-1244, which normally represses *SERPINE1* mRNA; consequently, derepression results in increased PAI-1 protein levels and the induction of cellular senescence [34].

Stress granules (SGs) promote the nuclear translocation of cyclin D1 and the phosphorylation of retinoblastoma protein (Rb) by recruiting PAI-1 to the cytoplasm and reducing its secretion, thereby sustaining cellular proliferation. Knockdown of the key SG structural protein G3BP1 or inhibition of SG formation via cycloheximide (CHX) reversed this stress-induced anti-senescence effect, whereas PAI-1 overexpression or exogenous supplementation reversed the senescent phenotype [59].

Notably, Eren et al. demonstrated that PAI-1 deficiency delays aging, preserves organ structural and functional integrity, and extends lifespan in klotho-knockout mice, underscoring the critical role of the serpin-regulated proteolytic cascade in vivo aging [60]. Conversely, loss-of-function mutations in the *SERPINE1* gene improve the cellular metabolic state, delay telomere shortening, and extend lifespan, reinforcing PAI-1 as a key aging marker from a reverse genetic perspective [61]. Pharmacological inhibition of PAI-1 with TM5441 significantly attenuates doxorubicin-induced cellular senescence through mechanisms including the suppression of reactive oxygen species (ROS) generation, the induction of antioxidant enzyme expression, and the inhibition of multiple aging-related signaling pathways [60].

### Therapeutic Targeting of PAI-1: From Preclinical Promise to Clinical Challenges

Given the pivotal role of PAI-1 in driving cellular senescence and age-related pathol ogies, pharmacological inhibition of PAI-1 has emerged as a promising therapeutic strategy. Unlike biological agents such as monoclonal antibodies, small-molecule inhibitors offer the advantage of oral bioavailability and better tissue penetration.

Early attempts to target PAI-1 focused on compounds like Tiplaxtinin (PAI-039), which inhibits PAI-1 activity by interfering with its vitronectin-binding site. While effective in preclinical models of thrombosis and fibrosis, its clinical utility was hampered by poor distinct specificity and potential bleeding risks due to interference with physiological hemostasis [75,76]. Consequently, the focus shifted towards developing specific inhibitors that do not compromise the basal coagulation cascade.

A significant breakthrough came with the development of the TM-series inhibitors, such as TM5275 and TM5441. These small molecules specifically bind to the “shutter region” of PAI-1, preventing it from transitioning into its active conformation or blocking its interaction with tPA/uPA without disrupting cell adhesion functions. TM5441, in particular, has demonstrated superior efficacy in preventing senescence. Mechanistically, it not only restores fibrinolytic activity but also suppresses SASP by inhibiting the TGF-β1 signaling pathway and reducing mitochondrial dysfunction [77]. As previously noted, TM5441 treatment significantly extends the lifespan of Klotho-deficient mice and preserves organ function, providing proof-of-concept for its anti-aging potential in vivo [60].

Despite these promising preclinical results, the translation of PAI-1 inhibitors to the clinic faces several hurdles. The primary concern remains the safety profile regarding hemostasis. Although TM-series inhibitors show a reduced risk of bleeding compared to tPA administration, the systemic inhibition of PAI-1 in elderly patients—who may have fragile vasculature or be on anticoagulants—requires rigorous safety evaluation. Furthermore, PAI-1 plays a physiological role in wound healing and cell migration; thus, chronic inhibition could theoretically impair tissue repair mechanisms. Currently, clinical insights are largely derived from epidemiological studies, such as those involving the Old Order Amish, where distinct loss-of-function mutations in *SERPINE1* are associated with longer telomeres and increased longevity [61]. Future research must focus on defining the precise therapeutic window and identifying biomarkers to stratify patients who would benefit most from anti-PAI-1 therapy. Crucially, insights from human genetics have provided validation for targeting PAI-1. While most human data linking PAI-1 to longevity are correlative, the Old Order Amish study provides rare genetic evidence supporting causality in humans. A landmark study of the Old Order Amish kindred revealed that individuals harboring a null mutation in *SERPINE1* exhibit longer telomeres, a lower prevalence of diabetes, and extended lifespans compared to unaffected kin. This “natural knockout” in humans strongly supports the hypothesis that PAI-1 inhibition could confer anti-aging and metabolic benefits in a clinical setting [61]. This “natural knockout” strongly suggests, but does not yet definitively prove, that pharmacological reduction in PAI-1 could recapitulate these benefits in the general population.

## 5. *SERPINE1* Gene and Cell Differentiation

### 5.1. Osteogenic Differentiation

The current evidence suggests that PAI-1 plays a seemingly paradoxical regulatory role in bone metabolism. E. Daci et al. reported that PAI-1 inactivation had a minimal effect on bone metabolism under physiological conditions in mice; however, in the absence of estrogen, PAI-1 deficiency prevented trabecular bone loss without affecting cortical bone [78]. Compared with wild-type controls, ovariectomized PAI-1 knockout mice presented increased trabecular bone volume but decreased bone formation rates. Furthermore, in a streptozotocin (STZ)-induced diabetic mouse model, PAI-1 contributed to bone mass loss and impaired fracture healing by inhibiting osteoblast differentiation [79]. Studies on type I diabetes-induced osteoporosis have demonstrated that PAI-1 deficiency selectively mitigated STZ-induced bone loss in female mice, revealing a sex-specific effect. In these female mice, PAI-1 deficiency attenuated diabetes-associated alterations in tibial Runx2, osterix, and alkaline phosphatase (ALP) levels, as well as serum osteocalcin, while also normalizing suppressed osteoclast numbers in the tibia. Notably, STZ markedly increased PAI-1 mRNA levels exclusively in the livers of female mice. In vitro, treatment with active PAI-1 inhibited osteogenic gene expression and mineralization in primary osteoblasts derived from female mouse calvaria [23].

Beyond metabolic bone loss, PAI-1 also significantly influences bone repair and structural development. Guangwen Jin et al. reported that the administration of a small molecule PAI-1 inhibitor (iPAI-1) significantly increased the trabecular bone volume in an estrogen deficiency osteoporosis model [80]. Additionally, PAI-1 depletion enhanced fracture callus size at days 7 and 14 postinjury by promoting extracellular matrix remodeling in a mouse femoral fracture model, with the enlarged callus rapidly normalizing in size and mineral content by day 21. The authors hypothesized that in female mice, type I diabetes impairs macrophage aggregation and phagocytosis during early bone repair through PAI-1, contributing to bone loss [81]. Moreover, Ishiwata et al. reported that increased expression of miR-224-5p in the rat lumbar spine at postnatal day 4 inhibited osteoblast differentiation by upregulating PAI-1, leading to vertebral malformation and segmentation defects characteristic of congenital kyphosis [82].

PAI-1 also participates in the pathological process of heterotopic ossification (HO). In a trauma-induced Achilles tendon HO model, PAI-1 deficiency markedly promoted HO and increased the number of ALP-positive cells in the tendon. Following trauma, inflammatory marker mRNA levels increase in both wild-type and PAI-1-deficient tendons, with PAI-1 mRNA also elevated in wild-type mice. PAI-1 deficiency significantly upregulated Runx2, osterix, and type I collagen expression in the Achilles tendons at 9 weeks post trauma. In vitro, PAI-1-deficient osteoblasts presented increased ALP activity and mineralization. Furthermore, during the differentiation of mouse adipose-derived stem cells into osteoblasts, PAI-1 deficiency increased ALP activity and osteocalcin expression while inhibiting chondrogenic differentiation. Collectively, these results indicate that PAI-1 deficiency promotes HO partly via enhanced osteoblast differentiation and ALP activity [83]. Additionally, Okada et al. demonstrated that dexamethasone (Dex) suppresses angiogenesis at injury sites during early bone repair, partially through PAI-1-mediated mechanisms [22]. Together, these findings suggest that PAI-1 deficiency is a key contributor to enhanced osteogenic differentiation and angiogenesis at sites of bone injury.

Conversely, several studies have identified PAI-1 overexpression as a critical promoter of osteogenesis. Nordstrom et al. reported that PAI-1 transgenic mice presented increased bone mineral density, biomechanical strength, and stiffness, particularly in females; these effects were primarily mediated by the binding affinity of PAI-1 to vitronectin [84]. Extending these findings to human physiology, our group previously demonstrated that human bone marrow mesenchymal stem cells (hBMSCs) from elderly donors display senescent phenotypes and markedly reduced osteogenic potential compared with those from young donors. Treatment with metformin (MF) dose-dependently restored the osteogenic capacity of aged hBMSCs (500–1000 μM), accompanied by significant downregulation of miR-181a-5p. The overexpression of miR-181a-5p inhibited osteogenesis, whereas its inhibition increased osteogenic gene expression. Bioinformatics analyses and dual-luciferase reporter assays confirmed that miR-181a-5p targets PAI-1; correspondingly, low PAI-1 expression impaired osteogenesis, whereas elevated PAI-1 promoted osteogenic differentiation in hBMSCs [24]. Similarly, Takafuji et al. examined the role of PAI-1 in mesenchymal stem cell differentiation into osteoblasts in wild-type and PAI-1 knockout mice. During MSC or mesenchymal ST-2 cell differentiation, the PAI-1 mRNA level progressively increased over time. Loss of PAI-1 significantly suppressed osteogenic gene expression, and siRNA-mediated knockdown of endogenous PAI-1 also inhibited osteoblast differentiation in vivo [85].

Further mechanistic insights suggest that PAI-1 may act via specific signaling cascades. Conditioned medium from PAI-1-transfected cells (P-CM) enhanced MSC osteogenesis in vitro, with hBMSCs treated with P-CM displaying increased osteogenic capacity relative to that of controls [86,87]. Osteogenesis is orchestrated by multiple transcription factors, including TGF-β, bone morphogenetic proteins (BMPs), Runx2, and osterix (OSX) [88]. BMP-2 is a potent osteogenic growth factor that promotes osteoblast differentiation and bone formation [89]. The indispensable role of Runx2 in osteogenesis, particularly in inducing major bone matrix genes in immature osteoblasts, was further demonstrated in mouse models in which Runx2 gene deletion resulted in bone formation defects [90]. Based on these specific molecular observations, we propose the following hypothesis regarding the mechanism of action: P-CM likely functions as an upstream activator of the BMP-2 signaling cascade. While direct binding interactions remain to be confirmed, the activation of the Smad1/5/8 axis strongly suggests that PAI-1-associated factors in the conditioned medium act downstream of BMP receptors to drive osteogenic gene transcription.

Finally, the protective role of PAI-1 extends to joint pathology. In an osteoarthritis (OA) model induced by combined ovariectomy (OVX) and medial meniscus destabilization (DMM), PAI-1 deficiency exacerbated subchondral bone mineral density (BMD) loss without significantly affecting articular cartilage degeneration. Mechanistically, PAI-1 exerts protective effects in OA by inhibiting IL-1β-induced RANKL expression and osteoclastogenesis, thereby maintaining bone metabolic homeostasis [91].

### 5.2. Adipogenic Differentiation

The plasminogen activation system and their primary inhibitor PAI-1 play a pivotal role in tissue regeneration by modulating ECM remodeling. Undifferentiated mouse embryonic stem cells (ESCs) exhibit minimal expression of PA system components; however, uPA activity transiently increases during differentiation, whereas tPA activity and PAI-1 protein levels peak at the terminal differentiation stage. Functional perturbations of the PA system via chemical inhibitors (amiloride, a uPA inhibitor) or genetic manipulation (induced overexpression of PAI-1) revealed that amiloride treatment or PAI-1 overexpression markedly suppressed the adipogenic differentiation capacity of ESCs. Conversely, knockout of PAI-1 in induced pluripotent stem cells (iPSCs) enhances their adipogenic potential. The PA system does not significantly influence ESC differentiation toward neural, myocardial, endothelial, or smooth muscle lineages [92]. Joji Kusuyama et al. demonstrated that the adipocytokine CXCL13 exacerbates the inflammatory phenotype during adipocyte differentiation, characterized by increased levels of the proinflammatory cytokines IL-6 and PAI-1 alongside decreased levels of the anti-inflammatory adipokine adiponectin. Targeted knockdown of the CXCL13 receptor CXCR5 by neutralizing antibodies or siRNA effectively attenuated the deferoxamine (DFO)-induced inflammatory response, suggesting that PAI-1 functions as an inflammatory mediator that inhibits adipocyte differentiation [93]. Similarly, Ola Hadadeh et al. reported that reduced PAI-1 expression promotes adipogenic differentiation in pluripotent ESCs, with PAI-1 knockdown in iPSCs leading to increased adipogenic capacity [92]. Furthermore, Jose M. Gallego-Escuredo et al. confirmed that PAI-1 acts as a proinflammatory cytokine that suppresses adipogenesis in the context of HIV-associated lipodystrophy [94].

### 5.3. Chondrogenic Differentiation

Studies investigating the role of PAI-1 in the chondrogenic differentiation of mesenchymal stem cells are limited. Previous research demonstrated that during in vitro differentiation of placental MSCs into chondrocytes and osteoblasts, the expression of silent information regulator 7 (SIRT7) was markedly decreased, accompanied by reduced levels of PAI-1, lamin A, and SM22α. Concurrently, the expression of the cell cycle regulator p16 and the tumor suppressor protein p53 was elevated, indicating that differentiated chondrocytes exhibited a premature senescence phenotype [95]. On the basis of these observations, it is hypothesized that decreased PAI-1 expression may suppress the osteogenic differentiation of MSCs while favoring chondrogenic lineage commitment. In support of this notion, a study examining arsenic exposure in humans revealed that arsenic-treated chondrocytes activated the mitogen-activated protein kinase (MAPK) signaling pathway, leading to the upregulation of cell cycle regulatory genes, including *CDKN1A*, *PAI-1*, *TP53*, *sequestosome-1* (*SQSTM1*), and the transcription factor *GATA-4*. This activation promoted chondrocyte senescence and accelerated articular cartilage aging in rat models [43]. These findings indirectly substantiate the inhibitory role of PAI-1 in chondrocyte differentiation.

### 5.4. Neurogenetic Differentiation

While the direct association between PAI-1 and neuronal cells remains incompletely understood, its roles in the fibrinolytic system and ECM regulation suggest that PAI-1 influences neuronal differentiation through indirect mechanisms. tPA has been demonstrated to participate in synaptic remodeling and nerve regeneration [96,97]. Notably, tPA expression and activity are markedly elevated in neural progenitor cells (NPCs) compared with mature neurons. Inhibition of tPA activity via PAI-1 overexpression or siRNA-mediated knockdown significantly impaired neurite outgrowth in NPCs, whereas tPA overexpression or exogenous supplementation promoted neurite extension. In vivo, the transplantation of wild-type NPCs into ischemic rat brains enhances axonal regeneration, an effect that is absent when NPCs derived from tPA knockout mice are used [97]. Furthermore, Soeda et al. reported that PAI-1 upregulates antiapoptotic genes (*Bcl-2*, *Bcl-XL*) and suppresses proapoptotic genes (*Bcl-Xs*, *Bax*) in neuronally differentiated PC-12 cells. By inhibiting mitochondrial apoptotic cascades—including cytochrome c release, caspase-3 activation, and DNA fragmentation—PAI-1 preserves neuronal morphology, suggesting that it promotes neural differentiation and survival. In Alzheimer’s disease (AD) models, PAI-1 confers neuroprotection against NMDA receptor-mediated excitotoxicity via the TGF-β1/Smad3 signaling pathway, whereas astrocyte-derived PAI-1 enhances PC-12 cell survival through the TrkA receptor and c-Jun/AP-1 pathway activation [98]. With respect to neuronal migration, Au et al. demonstrated that endothelial cell-secreted matrix calcium-binding protein (SPARC) and *SerpinE1* significantly facilitate interneuron migration in mouse medial ganglionic eminence explants and organotypic cultures [99]. In a human neuroblastoma SH-SY5Y cell model that differentiated into dopaminergic neuron-like cells, uPA promoted neuronal differentiation under low-serum conditions, whereas PAI-1, a uPA inhibitor, suppressed this differentiation, especially under lipopolysaccharide (LPS)-induced inflammatory conditions [100]. In stroke models, Adibhatla et al. reported that tPA facilitates early thrombolysis via plasmin activation but also activates MMP-9, contributing to neurotoxicity. Conversely, during later repair phases, tPA promotes vascular remodeling and nerve regeneration through vascular endothelial growth factor (VEGF)-mediated pathways, illustrating the temporal regulation of its effects [101]. Moreover, in fragile X syndrome (FXS) model mice (Fmr1 knockout), cultured NPCs exhibited tPA overexpression in glial cells, which contributed to aberrant neuronal migration. Neutralization of tPA by antibodies such as PAI-1 corrected excessive migration of doublecortin-positive neurons, promoted glial differentiation, and reduced the nuclear migration of radial glial cells, indicating that PAI-1 plays a crucial role in regulating glial-neuronal migration and differentiation [102]. Collectively, the role of PAI-1 in neuronal differentiation is context dependent and influenced by the cellular environment, disease state, and complex interplay within the fibrinolytic system alongside tPA and uPA, reflecting multifactorial regulatory mechanisms.

### 5.5. Fibroblast Differentiation

As a principal inhibitor of uPA and tPA, PAI-1 modulates ECM homeostasis by suppressing plasmin and MMP activities; however, its effects exhibit organ specificity and depend heavily on the pathological context. Under fibrotic conditions affecting the lung, liver, kidney, and skin, PAI-1 expression is markedly upregulated, leading to inhibited ECM degradation, as observed in models of bleomycin-induced pulmonary fibrosis and bile duct ligation-induced hepatic fibrosis [103,104,105,106,107,108]. TGF-β enhances PAI-1 expression via both Smad-dependent and non-Smad pathways, including ERK and JNK signaling, establishing a positive feedback loop that exacerbates ECM accumulation. PAI-1 deficiency mitigates tissue injury and fibrosis in several organs—such as the lung and kidney—potentially through activation of the plasmin/hepatocyte growth factor (HGF)/cyclooxygenase-2 (COX-2)/prostaglandin E2 (PGE2) axis, which inhibits collagen deposition [109]. Intriguingly, PAI-1 plays a dual regulatory role in cardiac fibrosis. While PAI-1 knockout reduces fibrosis following myocardial infarction [110], it paradoxically promotes spontaneous cardiac fibrosis in aged animals by inducing macrophage infiltration, MMP2/9 activation, and endothelial-to-mesenchymal transition (EndMT), culminating in collagen accumulation [111]. Consequently, therapeutic strategies targeting PAI-1—such as siRNA-mediated inhibition, the use of peroxisome proliferator-activated receptor gamma (PPARγ) agonists, or the modulation of downstream signaling pathways—have emerged as promising antifibrotic interventions [112,113].

## 6. Paradox and Association

### 6.1. Paradox in Aging

PAI-1 has been widely recognized as both a marker and a mediator of aging in numerous contemporary studies [8]. Notably, PAI-1 plays a dual role in pulmonary fibrosis, manifesting both pro-aging and antiaging effects, a phenomenon termed the “aging paradox.” A substantial body of evidence highlights the upregulation of PAI-1 during cellular senescence. For example, Kortlever et al. first demonstrated that the overexpression of PAI-1 alone is sufficient to induce stable cell cycle arrest in fibroblasts, even in the absence of p53 [114]. Subsequent investigations have confirmed the dual functionality of PAI-1 as both an aging biomarker and a prosenescent factor across various models, including vascular endothelial cells and keratinocytes, underscoring its pivotal role in cardiovascular aging.

Jiang et al. further clarified the bidirectional regulatory mechanism of PAI-1 in lung pathology. In a bleomycin-induced IPF mouse model, PAI-1 is markedly upregulated in type II alveolar epithelial cells (ATIIs), where it not only contributes to the promotion of cellular senescence but also may facilitate fibrosis resolution during disease recovery [38]. Similarly, partial deletion of PAI-1 in a kl/kL premature aging mouse model significantly extends the median lifespan and preserves telomere length, suggesting its therapeutic potential in mitigating aging under certain conditions [60]. Moreover, PAI-1 is considered a candidate target within the SASP secretome, and its inhibition shows promise in alleviating age-related pathologies such as emphysema [115]. PAI-1 deficiency has also been shown to normalize key SASP factors—including IL-6 and IGFBP-3—in kl/kL mice, indicating that these molecules act as downstream regulatory nodes in aging-related signaling pathways [116].

At the signaling level, Rana et al. demonstrated that TGF-β induces PAI-1 expression through the activation of multiple pathways, thereby driving cellular senescence [26]. Intriguingly, PAI-1 exhibits mechanistic duality depending on the cellular context. In primary mouse ATII cells and A549 cells, PAI-1 associates with the proteasome complex to inhibit p53 degradation, thereby inducing senescence. Furthermore, PAI-1 overexpression can trigger ATII cell senescence via p53-independent mechanisms [117]. Conversely, silencing PAI-1 significantly suppresses adriamycin- and bleomycin-induced senescence and p53 upregulation in rat lung epithelial cells [38].

### 6.2. Paradox in Cell Differentiation

#### 6.2.1. Paradox in Osteogenic Differentiation 

**Figure 2:**
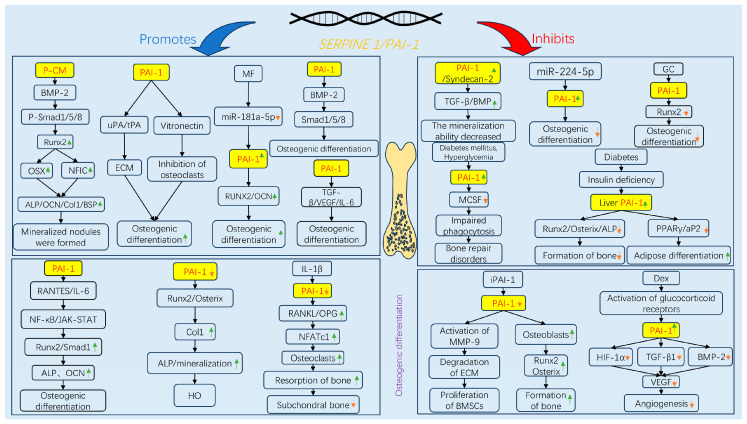
Signaling pathways governing the dual regulatory role of PAI-1 in osteogenic differentiation. The schematic illustrates the context-dependent mechanisms by which PAI-1 either promotes (**left panel**, blue arrows) or inhibits (**right panel**, red arrows) osteogenesis. (**Left Panel**) PAI-1 promotes osteogenesis primarily through the BMP-2/Smad signaling pathway (activated by PAI-1 conditioned medium, P-CM) and by stabilizing ECM via inhibition of uPA/tPA-mediated proteolysis. Additionally, Metformin (MF) enhances osteogenesis by downregulating miR-181a-5p, thereby relieving the suppression of PAI-1.(**Right Panel**) Conversely, under pathological conditions such as diabetes (hyperglycemia), glucocorticoid (GC/Dex) administration, or aging, elevated PAI-1 levels inhibit key osteogenic transcription factors (Runx2, Osterix) and promote adipogenesis (via PPARγ). Pharmacological inhibition of PAI-1 (e.g., iPAI-1) or genetic knockdown restores osteoblast differentiation by activating MMP-9 and enhancing Runx2 expression. Abbreviations: ALP, alkaline phosphatase; BMP-2, bone morphogenetic protein-2; BSP, bone sialoprotein; Col1, type I collagen; Dex, dexamethasone; ECM, extracellular matrix; GC, glucocorticoids; HIF-1α, hypoxia-inducible factor-1alpha; HO, heterotopic ossification; iPAI-1, small molecule PAI-1 inhibitor; JAK-STAT, Janus kinase-signal transducer and activator of transcription; MCSF, macrophage colony-stimulating factor; MF, metformin; MMP, matrix metalloproteinase; NFATc1, nuclear factor of activated T-cells, cytoplasmic 1; NFIC, nuclear factor I C; NF-κB, nuclear factor kappa-light-chain-enhancer of activated B cells; OCN, osteocalcin; OPG, osteoprotegerin; OSX, osterix; P-CM, PAI-1 transfected cell-conditioned medium; PPARγ, peroxisome proliferator-activated receptor gamma; RANKL, receptor activator of nuclear factor kappa-B ligand; RANTES, regulated on activation, normal T cell expressed and secreted; Runx2, Runt-related transcription factor 2; TGF-β, transforming growth factor-beta; tPA, tissue-type plasminogen activator; uPA, urokinase-type plasminogen activator; VEGF, vascular endothelial growth factor.

Accumulating evidence indicates that PAI-1 plays a dual role in regulating osteogenic differentiation, reflecting a complex and “paradoxical” biological function (Figure 2). On the one hand, substantial in vitro and in vivo evidence supports the inhibitory role of PAI-1. Established research has demonstrated that PAI-1 knockout mice display enhanced osteogenic capacity, reduced bone loss, increased trabecular bone volume, or elevated HO in models of estrogen deficiency [78], diabetes [79], trauma repair [81], and spinal dysplasia [82]. This improved bone metabolism is associated primarily with the upregulation of key osteogenic factors such as Runx2, Osterix, and ALP following PAI-1 deficiency [83] and may also indirectly influence osteogenesis via the modulation of inflammatory responses, macrophage recruitment, and angiogenesis [22]. Moreover, exogenous active PAI-1 directly inhibits the expression of osteogenesis-related genes and mineralization in primary osteoblasts, further supporting its inhibitory potential under certain pathological conditions [23]. Conversely, PAI-1 has also been reported to exert a bone-promoting effect. Transgenic mice overexpressing PAI-1 exhibit significant increases in bone mineral density and mechanical strength, particularly in females, an effect attributed to the interaction of PAI-1 with vitronectin [84]. Our previous work demonstrated that PAI-1 is a direct target of miR-181a-5p, which is upregulated in senescent human bone marrow mesenchymal stem cells and suppresses PAI-1 expression, leading to diminished osteogenic differentiation. Inhibition of miR-181a-5p or direct upregulation of PAI-1 significantly enhances the osteogenic potential of hBMSCs [24]. Additionally, Takafuji et al. reported a gradual increase in PAI-1 expression during MSC differentiation into osteoblasts, with PAI-1 deletion or siRNA-mediated knockdown markedly impairing osteogenic gene expression [85]. In vitro, conditioned medium from PAI-1-transfected cells activates the BMP-2/Smad-1/5/8 pathway and induces Runx2 and Osterix expression, thereby promoting hBMSC osteogenesis [86].

#### 6.2.2. Fibrosis

This “paradox” of differentiation also extends to fibrosis. While PAI-1 functions as a potent profibrotic driver in most tissues, its role is strictly context-dependent.

In the lung, mechanistic studies utilizing the bleomycin-induced injury model have established that PAI-1 is critical for fibrosis development. PAI-1-deficient (PAI-1−/−) mice exhibit significantly attenuated collagen deposition and reduced mortality compared to wild-type (PAI-1+/+) mice following bleomycin challenge. Conversely, PAI-1 overexpression exacerbates the fibrotic phenotype. Pharmacological inhibition with small molecules such as TM5275 and Tiplaxtinin has been shown to effectively suppress plasminogen activator inhibitory activity, thereby ameliorating pulmonary fibrosis [19,118].

Similarly, in renal pathology, PAI-1 mediates fibrosis in models of unilateral ureteral obstruction (UUO) and diabetic nephropathy. PAI-1−/− mice are protected against interstitial fibrosis and macrophage infiltration compared to their wild-type counterparts. This profibrotic effect is largely dependent on the inhibition of ECM degradation and the promotion of epithelial–mesenchymal transition (EMT). Consequently, PAI-1 inhibitors (e.g., TM5441) have demonstrated efficacy in preserving renal structure and function in preclinical models [119,120].

In hepatic fibrosis induced by bile duct ligation or carbon tetrachloride (CCl4), PAI-1 levels are markedly elevated. PAI-1 deficiency or inhibition accelerates the resolution of liver fibrosis by enhancing proteolytic activity and modulating hepatic stellate cell activation [121].

The notable exception to this pattern is cardiac fibrosis, where PAI-1 deficiency paradoxically exacerbates fibrotic progression. In aged mice, complete PAI-1 deficiency (PAI-1−/−) results in spontaneous cardiac fibrosis compared to age-matched wild-type controls. This has been attributed to the loss of PAI-1-mediated regulation of integrin αvβ3. This dysregulation leads to increased vascular permeability, local inflammation, excessive extracellular matrix remodeling, and augmented TGF-β signaling, a potent profibrotic driver [122,123].

#### 6.2.3. Neuronal Differentiation

PAI-1 plays a complex, dual regulatory role in the nervous system, which is strictly dependent on the stage of neuronal maturation and the temporal phase of injury.

First, regarding NPCs, PAI-1 primarily acts as a negative regulator of neurogenesis and neurite outgrowth. During development and regeneration, tPA is essential for degrading the extracellular matrix to facilitate neurite extension and migration. PAI-1 inhibits this process by suppressing tPA proteolytic activity. Studies indicate that in NPCs, PAI-1 overexpression or knockdown significantly impairs or enhances neurite outgrowth, respectively, thereby acting as a brake on neuronal differentiation [96,100].

In contrast, in mature neurons, PAI-1 can exert neuroprotective effects under specific stress conditions. In PC-12 cells and mature neuronal models, PAI-1 has been shown to upregulate antiapoptotic factors (Bcl-2, Bcl-XL) while downregulating proapoptotic genes (Bax, Bcl-Xs), thereby inhibiting mitochondrial apoptosis [100]. Furthermore, in the context of neurodegenerative pathology such as Alzheimer’s disease, PAI-1 protects mature neurons from excitotoxicity via the TGF-β1/Smad3 and TrkA/c-Jun pathways [100], and modulates aberrant migration in fragile X syndrome models [102].

Crucially, the function of PAI-1 diverges significantly between the acute injury phase and the subacute repair phase. In the acute phase of injury (e.g., ischemic stroke or excitotoxicity), PAI-1 may offer protection by inhibiting tPA-mediated neurotoxicity and BBB integrity. Excessive tPA activity acutely can exacerbate neuronal death via NMDA receptor activation; thus, PAI-1 serves as a protective shield [35]. However, during the repair and remodeling phase, PAI-1 becomes detrimental. Successful recovery requires synaptic plasticity and axonal regeneration, processes that depend on tPA activity. Persistently elevated PAI-1 levels in the chronic phase inhibit these regenerative mechanisms, thereby impeding functional recovery [124].

Collectively, these findings illustrate that PAI-1′s impact is not uniform but is determined by the specific cell type and the pathological timing.

#### 6.2.4. Integrative Hypothesis: The Determinants of PAI-1 Bidirectionality

To reconcile these apparent contradictions across osteogenesis, fibrosis, and neuro genesis, we propose an integrative hypothesis: PAI-1 functions not merely as a protease inhibitor but as a spatiotemporal “molecular switch” whose net effect is determined by three critical variables. 

The biological outcome of PAI-1 signaling depends heavily on its binding partners within the niche. For instance, in bone, PAI-1’s interaction with vitronectin can stabilize the extracellular matrix to support osteoblast attachment, whereas its interaction with uPAR/integrins in a distinct inflammatory milieu may suppress differentiation signaling. This explains why PAI-1 drives fibrosis in the lung but paradoxically protects against fibrosis in the heart [117,118].

The timing of PAI-1 expression dictates its function. Acute upregulation of PAI-1 following injury is often cytoprotective. However, sustained, chronic overexpression—typical of aging or metabolic disease—shifts its role towards inhibition of repair, blocking the necessary matrix remodeling required for tissue regeneration and neurite outgrowth [18].

Physiological levels of PAI-1 are essential for homeostasis. In contrast, the “SASP-associated” supraphysiological levels secreted by senescent cells act in an autocrine/paracrine loop to lock cells in a specific state, effectively overriding normal differentiation cues [125,126].

**Figure 3:**
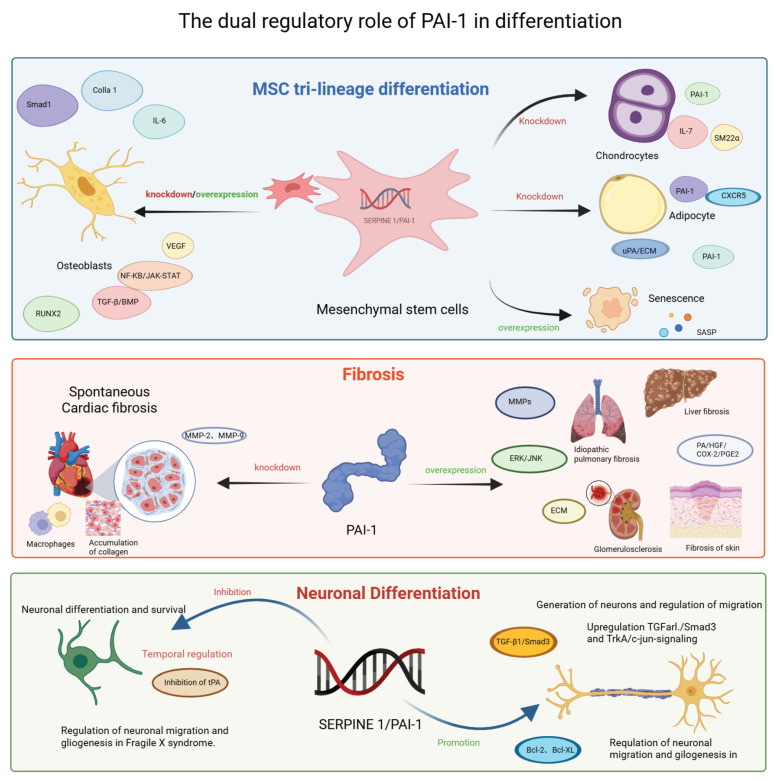
Context-dependent modulation of stem cell differentiation, fibrosis and Neuronal Differentiation by SERPINE1/PAI-1. This schematic summarizes the pleiotropic roles of PAI-1 across three distinct biological processes. (**Top Panel**) MSC Tri-lineage Differentiation: PAI-1 regulates the fate of MSCs. Knockdown or inhibition generally favors chondrogenesis and adipogenesis, whereas PAI-1 overexpression is linked to cellular senescence and SASP. Its role in osteogenesis is complex and context-dependent. (**Middle Panel**) Fibrosis: PAI-1 acts as a profibrotic driver in the liver, lung, kidney, and skin, promoting ECM accumulation via MMP inhibition and ERK/JNK signaling. Paradoxically, in the heart, PAI-1 deficiency exacerbates spontaneous cardiac fibrosis. (**Bottom Panel**) Neuronal Differentiation: PAI-1 exerts a dual effect on neurons. It can inhibit neurite outgrowth and migration by suppressing tPA activity but also promotes neuronal survival and gliogenesis via TGF-β1/Smad3 and anti-apoptotic (Bcl-2) pathways in specific contexts such as Fragile X syndrome. Abbreviations: Bcl-2, B-cell lymphoma 2; Bcl-XL, B-cell lymphoma-extra large; BMP, bone morphogenetic protein; COX-2, cyclooxygenase-2; CXCR5, C-X-C motif chemokine receptor 5; ECM, extracellular matrix; ERK, extracellular signal-regulated kinase; HGF, hepatocyte growth factor; IL, interleukin; JNK, c-Jun N-terminal kinase; MMP, matrix metalloproteinase; NF-κB, nuclear factor kappa-light-chain-enhancer of activated B cells; PA, plasminogen activator; PGE2, prostaglandin E2; RUNX2, Runt-related transcription factor 2; SASP, senescence-associated secretory phenotype; SM22α, smooth muscle protein 22-alpha; TGF-β, transforming growth factor-beta; tPA, tissue-type plasminogen activator; TrkA, tropomyosin receptor kinase A; uPA, urokinase-type plasminogen activator; VEGF, vascular endothelial growth factor.

To better understand the role of PAI-1 in osteogenic differentiation, we performed a comparative analysis of high-quality studies (Table 2) and summarized the schematic diagram of PAI-1 promoting/inhibiting differentiation (Figure 3). These studies span diverse animal disease models and elucidate key pathways from robust literature, shedding new light on the multifaceted functions of PAI-1. The dual “inhibitory and promotive” roles of PAI-1 in cell differentiation appear to be modulated by microenvironmental factors, the inflammatory status, and stem cell senescence. This paradox underscores the significant context-dependence of the biological activities of PAI-1. Future research must further delineate the upstream and downstream signaling networks, as well as the spatiotemporal expression patterns of PAI-1 in specific pathological contexts, providing a theoretical basis for targeted interventions in complex age-related diseases.

### 6.3. Mechanistic Framework: Distinguishing PAI-1 Roles in Aging Versus Differentiation

The induction of cell differentiation by PAI-1 may represent a pathway linking cell maturation and aging, yet the mechanisms driving these two outcomes act through distinct logical distinct pathways. To clarify the interpretability of these pleiotropic effects, we propose a mechanistic framework distinguishing where the evidence is robust versus where it remains context-dependent.

Substantial evidence confirms that PAI-1 drives senescence primarily through intracellular signaling and metabolic stress. It is well established that PAI-1 interacts with the proteasome to inhibit p53 degradation. This triggers the p53/p21 pathway, leading to irreversible cell cycle arrest [32]. As demonstrated in our previous studies, osteogenic differentiation requires increased mitochondrial activity. However, excessive ROS accumulation activates the DNA damage response (DDR), which converges on PAI-1 upregulation. Here, PAI-1 acts as a downstream effector of metabolic stress, locking cells in a senescent state via the p53 pathway [40].Definitive evidence shows that PAI-1 is a key component of SASP. Elevated PAI-1 in aged MSCs creates a repressive microenvironment that inhibits stem cell self-renewal and suppresses osteogenic differentiation while promoting adipogenesis via TGF-β signaling [127].

In contrast to the universal p53-mediated senescence mechanism, the role of PAI-1 in differentiation appears to be more speculative and dependent on specific spatiotemporal contexts. Emerging evidence suggests that lineage-specific differentiation cues can antagonize aging. For instance, metformin was reported to upregulate PAI-1 via the suppression of miR-181a-5p, thereby fostering osteogenic differentiation while concomitantly attenuating SA-β-gal activity [24]. This implies that under specific therapeutic contexts, PAI-1 induction serves as a differentiation signal rather than an aging driver. We postulate that the paradoxical functions of PAI-1 result from the timing of its expression. In early osteogenesis, BMP-2-induced phosphorylation of Smad1/5/8 recruits histone acetyltransferases to the PAI-1 promoter, transiently increasing transcription to facilitate lineage commitment. Conversely, at later stages, DNMT3b-mediated hypermethylation suppresses PAI-1 to prevent premature senescence [22]. This proposed “epigenetic switch” mechanism awaits definitive experimental validation but offers a logical explanation for the observed dual effects. Recent studies propose a post-transcriptional mechanism where SGs sequester PAI-1 mRNA during acute stress to preserve proliferation, but release it to induce senescence during prolonged stress [59].

**Figure 4:**
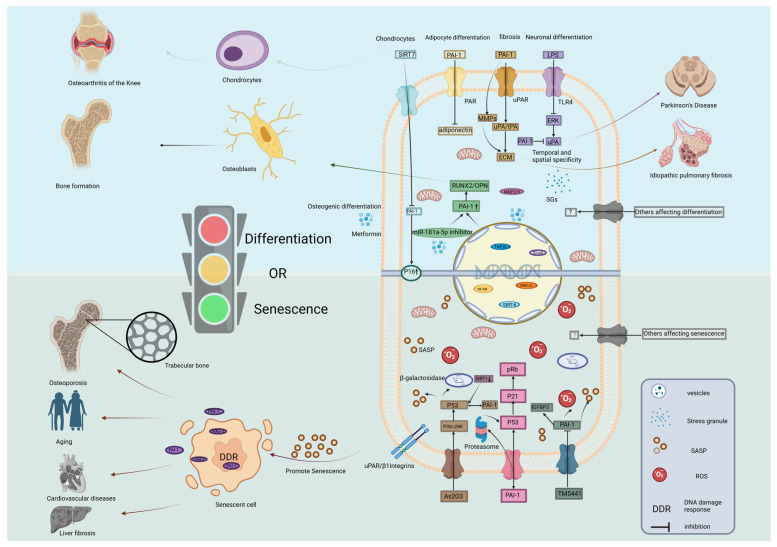
Molecular mechanisms underlying the role of SERPINE1/PAI-1 as a switch between cell differentiation and senescence. The schematic illustrates the intracellular signaling cascades by which PAI-1 directs cell fate. (**Bottom Panel**) Senescence Pathways: PAI-1 promotes cellular senescence through multiple mechanisms: (i) The P53/P21/pRb pathway and P16 activation trigger cell cycle arrest; (ii) PAI-1 can act on P53 through proteasome to stabilize it and interact with P53. (iii) IGFBP3 is downstream of PAI-1, pharmacological agents like TM5441 inhibit PAI-1. (iv) As2O3 acts on P53 through P38/JNK signaling to produce SASP such as β-galactosidase. (v) Accumulation of ROS and DDR promote the release of SASP, leading to osteoporosis, cardiovascular disease, and liver fibrosis. (**Top Panel**) Differentiation Pathways: PAI-1 regulates differentiation via distinct routes: (i) Modulation of PAR signaling affects adipocyte differentiation; (ii) Regulation of the MMPs and uPA/tPA signaling pathways by PAI-1 is important for the ECM, leading to pulmonary fibrosis. (iii) The TLR4/ERK pathway is implicated in neural differentiation (e.g., Parkinson’s disease context); (iv) In osteoblasts, Metformin downregulates miR-181a-5p, relieving the suppression of PAI-1 and enhancing RUNX2/OPN expression. SIRT6 also modulates these epigenetic landscapes. (v) SIRT7 upregulates P16 by inhibiting PAI-1 and affects the differentiation of chondrocytes. The “Traffic Light” represents the critical decision node where PAI-1 levels determine the shift between differentiation (Green/Go) and senescence (Red/Stop). Abbreviations: As2O3, arsenic trioxide; DDR, DNA damage response; ECM, extracellular matrix; ERK, extracellular signal-regulated kinase; JNK, c-Jun N-terminal kinase; LPS, lipopolysaccharide; MMP, matrix metalloproteinase; NF-κB, nuclear factor kappa-light-chain-enhancer of activated B cells; OPN, osteopontin; PAR, protease-activated receptor; Rb, retinoblastoma protein; ROS, reactive oxygen species; RUNX2, Runt-related transcription factor 2; SASP, senescence-associated secretory phenotype; SIRT, sirtuin; TLR4, Toll-like receptor 4; uPA, urokinase-type plasminogen activator; uPAR, urokinase plasminogen activator receptor. Created in BioRender. Ke, J. (2025) https://BioRender.com/e8qwrd2.

Collectively, these observations support a model where PAI-1 functions as a “molecular switch” (Figure 4). The distinction lies in the duration and localization. Transient, epigenetically regulated PAI-1 expression (via promoter methylation or microRNA modulation) facilitates differentiation by remodeling the ECM and modulating specific signaling nodes such as BMP-2. Conversely, sustained PAI-1 accumulation—driven by genetic polymorphisms (e.g., 4G allele) or chronic metabolic stress—overwhelms these checkpoints, activating the p53 axis and driving pathological aging [27].

## 7. Summary and Outlook

As a key aging marker gene, PAI-1 has been extensively validated for its association with aging and various degenerative diseases. Both clinical and experimental studies have demonstrated that PAI-1 levels markedly increase with advancing age and contribute to the pathogenesis of multiple age-related disorders, including atherosclerosis, diabetic nephropathy, and osteoporosis. Despite the well-established role of PAI-1 as a biomarker of aging, its precise mechanisms remain inadequately defined, particularly within diverse tissue microenvironments, spatiotemporal contexts, and disease models. Moreover, the regulatory networks and downstream effectors of PAI-1 have yet to be fully elucidated. Recently, increasing evidence has revealed the pivotal role of PAI-1 in regulating cell differentiation. The “duality” of PAI-1 in mediating both differentiation and senescence may represent a critical regulatory node governing cell fate decisions—regarding proliferation, differentiation, or senescence—thus underscoring its complexity in modulating the destiny of pluripotent stem cells.

From a therapeutic perspective, the identification of PAI-1 as a “molecular switch” opens new avenues for pharmacological intervention. Preclinical studies using small-molecule inhibitors, such as TM5441, have demonstrated causal efficacy in attenuating cellular senescence and mitigating fibrosis in rodent models. However, it is important to note that these findings are largely derived from experimental models. Whether PAI-1 inhibition will translate into clinical efficacy for human aging-related diseases remains a hypothesis that requires validation in rigorous clinical trials. The primary limitation lies in the need to balance the inhibition of pathological PAI-1 activity with the preservation of its physiological role in hemostasis and wound healing. Furthermore, given the pleiotropic effects of PAI-1, systemic inhibition could yield unintended consequences in non-target tissues. Therefore, future therapeutic strategies must focus on developing delivery systems with high tissue specificity and identifying biomarkers to stratify patients who would benefit most from PAI-1 inhibition.

Currently, the detailed mechanisms underlying the regulatory effects of PAI-1 on aging and differentiation, including its spatiotemporal expression patterns, remain unclear. Potential regulatory modalities, such as miRNA interactions, DNA methylation, and histone acetylation, warrant further investigation. Beyond mechanistic studies, our research group is actively exploring the translational potential of modulating PAI-1 dynamics. By deciphering how PAI-1 determines the balance between stem cell differentiation and senescence, we aim to provide the theoretical foundation for precision medicine approaches that target PAI-1 to treat chronic age-related diseases while minimizing adverse effects.

## Figures and Tables

**Table 1:** Association of PAI-1 with a variety of aging-related diseases.

Disease	Study Type	Proposed Mechanism/Key Findings	Ref.
Idiopathic pulmonary fibrosis	Preclinical (In vivo): mice	PAI-1 mediates TGF-β1-induced alveolar type II cell senescence and senescence-associated secretory phenotype (SASP) secretion through upregulating p16, thereby promoting profibrotic macrophage activation.	[26]
Cardiovascular diseases	Preclinical (In vivo): Mouse models	PAI-1 reduces infarct volume in permanent stroke by inhibiting t-PA neurotoxicity but exacerbates injury in thrombotic stroke by blocking t-PA-mediated thrombolysis and delaying spontaneous reperfusion.	[35]
Diabetes mellitus type2	Clinical (Observational)	Elevated PAI-1 secretion by ADSC from CAD and CAD + T2DM patients impairs angiogenic activity by disrupting the balance between pro- and anti-angiogenic factors, despite increased production of pro-angiogenic growth factors.	[36]
Idiopathic pulmonary fibrosis	Preclinical (In vitro)	PAI-1 binds to proteasome components, inhibits proteasome activity and p53 degradation, thereby increasing p53 expression and promoting ATII cell senescence.	[37]
Idiopathic pulmonary fibrosis	Preclinical (In vitro): Rat ATII (L2) cells; Preclinical (In vivo): mouse model	PAI-1 induces ATII cell senescence by activating the p53-p21-Rb cell cycle repression pathway, contributing to lung fibrosis development.	[38]
Aging-related susceptibility to lung fibrosis	Preclinical (In vivo): C57BL/6 mice and Preclinical (In vitro): CCL-210 cell line	PAI-1 expression increases with age in lung fibroblasts, protecting them from apoptosis and enhancing their sensitivity to TGF-β1, thereby promoting aging-related susceptibility to lung fibrosis.	[39]
Idiopathic pulmonary fibrosis	Preclinical (In vitro)	Increased PAI-1 expression contributes to the apoptosis paradox in idiopathic pulmonary fibrosis (IPF) by dichotomously regulating p53 to promote ATII cell apoptosis while protecting fibroblasts.	[40]
Glomerulosclerosis	Clinical (Observational)	Glomerular endothelial cell senescence drives age-related kidney disease through PAI-1-mediated podocyte apoptosis and detachment.	[41]
Aging-related bone metabolic disruption	Preclinical (In vitro)	SIRT6 deficiency increases PAI-1 expression, which drives osteocyte senescence and upregulates Sost and Fgf23, leading to low-turnover osteopenia in aged mice.	[42]
Osteoarthritis	Preclinical (In vitro): Human articular chondrocytes (HC-a); Preclinical (In vivo): Male Wistar rats	Arsenic induces chondrocyte senescence via p38/p16 and JNK/p53-p21 signaling pathways, activating GATA4-NF-κB to trigger SASP secretion that includes PAI-1, thereby accelerating articular cartilage aging and degeneration.	[43]
Human osteosarcoma	Clinical (Observational)	PAI-1, a direct target of miR-143, promotes osteosarcoma invasion and lung metastasis by upregulating MMP-13 expression and secretion.	[44]
Ovarian cancer	Preclinical (In vitro): ES-2, JHOC-9, JHOC-5, SKOV3, JHOC-7, JHOC-8	PAI-1 plays a pro-proliferation and anti-apoptosis role in ovarian cancer cells by regulating G2/M cell cycle progression and inhibiting mitochondria-mediated intrinsic apoptosis pathway.	[45]
Breast cacer	Clinical (Observational)	Elevated pre-surgical plasma PAI-1 reflects tumor-driven dysregulation of fibrinolysis and extracellular matrix remodeling, serving as an independent prognostic biomarker for reduced relapse-free survival in breast cancer patients.	[46]
Psoriatic skin and in basal cell carcinomas	Clinical (Observational)	PAI-1 is upregulated in psoriatic epidermis and in the peritumoral stroma of basal cell carcinoma, where it contributes to tissue remodeling, angiogenesis, and may modulate tumor cell invasion.	[47]
Head and neck carcinoma	Clinical (Observational)	*SERPINE1*/PAI-1 promotes HNSCC progression by activating PI3K/AKT signaling to enhance cell migration and confer cisplatin resistance, thereby leading to poor clinical outcomes.	[48]
Triple-negative breast cancer	Clinical (Observational)	PAI-1 secreted by TNBC cells stimulates endothelial CCL5 expression, which acts via CCR5 in a paracrine manner to enhance TNBC cell invasion and metastasis while forming a positive feedback loop by further inducing PAI-1 secretion.	[49]
werner syndrome	Preclinical (In vitro): Primary human diploid fibroblasts (HDF)	PAI-1 is markedly overexpressed (>1000-fold) in prematurely senescent Werner syndrome fibroblasts, where it dysregulates fibrinolysis and drives a pro-coagulant, pro-fibrotic extracellular matrix remodeling that contributes to cellular growth arrest and age-related pathology.	[50]
Atherosclerosis	Preclinical (In vitro): HUVECs	PAI-1 is constitutively upregulated in senescent endothelial cells via IL-1α–mediated signaling, representing a cell-type–specific mechanism that distinguishes endothelial senescence from fibroblast aging.	[51]
Atherosclerosis	Preclinical (In vitro): HUVECs	Homocysteine upregulates PAI-1 expression, which accelerates endothelial senescence.	[52]
Cardiovascular diseases	Preclinical (In vivo): C57Bl/6 wildtype mice	PAI-1 contributes to homocysteine-induced endothelial senescence by upregulating key senescence regulators (integrin β3, p16, p53, p21) and suppressing antioxidant defense mechanisms (Nrf2, catalase)	[53]
venous thrombosis	Preclinical (In vivo): C57BL/6 mice	Aging impairs venous thrombus resolution by increasing active plasma PAI-1, which inhibits tPA/uPA-mediated fibrinolysis, thereby reducing thrombolytic capacity and promoting larger thrombus formation.	[54]
Atherosclerosis	Preclinical (In vitro): HUVECs	Replicative senescence upregulates PAI-1 expression in endothelial cells, which is associated with increased expression of adhesion molecules (ICAM1, SELE, CCL2, integrins) and enhanced monocyte adhesion, thereby promoting atherosclerotic pathogenesis.	[55]
Senescence	Preclinical (In vitro): HUVECs	Sirt1 inhibition induces premature endothelial senescence by hyperacetylating p53, which transcriptionally upregulates PAI-1 expression and downregulates eNOS, creating a pro-senescent, pro-thrombotic phenotype.	[56]
Diabetes	Preclinical (In vivo): mice	Endothelium-specific SIRT1 overexpression inhibits hyperglycemia-induced vascular cell senescence by attenuating oxidative stress, which suppresses the upregulation of PAI-1 and other senescence-associated markers.	[57]
Senescence	Preclinical (In vitro): Human breast cancer cell lines (MCF-7, ZR-75-1), human primary fibroblasts (IMR-90), retinal pigment epithelial cells (ARPE-19), and 293T cells.	PAI-1 acts as a secreted mediator of stress-induced senescence by inhibiting t-PA-mediated proteolysis of IGFBP3, thereby protecting IGFBP3 from degradation and allowing it to accumulate and induce cellular senescence.	[58]
Senescence	Preclinical (In vitro)	Stress granules counteract cellular senescence by sequestering PAI-1, thereby preventing its secretion and relieving its inhibition of cyclin D1 nuclear translocation and RB phosphorylation to maintain a proliferative state.	[59]
Senescence	Preclinical (In vivo): mice	PAI-1 drives premature aging in Klotho-deficient mice by inhibiting t-PA–mediated proteolysis of IGFBP-3, thereby promoting cellular senescence, tissue dysfunction, and reducing lifespan.	[60]
Senescence	Clinical (Observational)	PAI-1 functions as a critical mediator of cellular senescence and biological aging; *SERPINE1* null mutation-mediated reduction in PAI-1 protects against age-related telomere attrition and metabolic dysfunction, thereby extending human lifespan.	[61]

**Table 2:** PAI-1 promotes/inhibits osteogenesis.

Models of Disease	Study Type	Proposed Mechanism/Key Findings	Promotes/Inhibits	Ref.
Angiogenesis during bone repair in mice	Preclinical (In vivo)	Glucocorticoid (dexamethasone)-induced decreases in angiogenesis during early bone repair are mediated partly by PAI-1, which suppresses the expression of VEGF, HIF-1α, TGF-β1, and BMP-2 at the injury site.	Inhibition	[22]
Diabetes in female mice	Preclinical (In vivo and in vitro): wild-type mice	PAI-1 contributes to diabetic osteoporosis in female mice by impairing osteoblast differentiation and mineralization, suppressing osteoclast formation, and promoting adipogenesis in bone tissue.	Inhibition	[23]
A bone defects rat model	Preclinical (In vitro and in vivo)	Metformin enhances osteogenic differentiation of aging mesenchymal stem cells by downregulating miR-181a-5p, which relieves its inhibitory effect on PAI-1, thereby increasing PAI-1 expression and promoting bone formation.	Promotion	[24]
Female mice	Preclinical (In vivo)	PAI-1 deficiency protects against trabecular bone loss induced by estrogen deficiency, indicating that PAI-1 promotes bone resorption in estrogen-deficient conditions.	Inhibition	[78]
Diabetes in female mice	Preclinical (In vivo)	AI-1 contributes to impaired bone repair in diabetic female mice by suppressing osteoblast differentiation and reducing the number of ALP-positive osteoblastic cells at the injury site.	Inhibition	[79]
Murine estrogen deficiency-induced osteoporosis model	Preclinical (In vivo)	Inhibition of PAI-1 by a small-molecule iPAI-1 stimulates bone formation through increasing the number of bone marrow stromal cells and enhancing osteoblast precursor differentiation, while also partially suppressing osteoclast activity, thereby preventing ovariectomy-induced bone loss.	Inhibition	[80]
Diabetic female mice	Preclinical (In vivo and in vitro)	PAI-1 contributes to delayed bone repair in diabetic female mice by reducing macrophage accumulation and phagocytic activity at the injury site, thereby impairing the early inflammatory phase of bone healing.	Inhibition	[81]
The lumbar spine of a rat model of congenital kyphoscoliosis	Preclinical (In vivo): rat model	Upregulated miR-224-5p increases the expression of Pai-1, which in turn suppresses osteoblast differentiation.	Inhibition	[82]
Heterotopic Ossification Induced by Achilles Tenotomy in Thermal Injured Mice	Preclinical (In vivo and in vitro)	Endogenous PAI-1 plays a protective role against heterotopic ossification by suppressing osteoblast differentiation, ALP activity, and mineralization, thereby limiting ectopic bone formation following trauma and inflammation.	Inhibition	[83]
Female mice	Preclinical (In vivo): Transgenic mouse model	PAI-1 over-expression increases bone strength and mineralization in an age-dependent and gender-specific manner primarily through its vitronectin-binding ability	Promotion	[84]
PAI-1 KO mice	Preclinical (In vitro and In vivo)	PAI-1 deficiency suppresses osteoblastic differentiation of mesenchymal stem cells independently of the fibrinolytic system and Smad phosphorylation	Promotion	[85]
Calvarial defects mice	Preclinical (In vitro and in vivo)	P-CM promotes osteogenic differentiation of hBMSCs by upregulating NFIC and OSX through the activation of Smad-1/5/8 signaling	Promotion	[86]
OA model mice	Preclinical (In vivo and in vitro)	PAI-1 deficiency exacerbates subchondral osteopenia in osteoarthritis by enhancing IL-1β-induced RANKL expression in osteoblasts and promoting osteoclast formation, thereby increasing bone resorption.	Promotion	[91]

## Data Availability

No new data were created or analyzed in this study. Data sharing is not applicable to this article.

## References

[1] Shaikh S.B., Balaya R.D.A., Dagamajalu S., Bhandary Y.P., Unwalla H., Prasad T.S.K., Rahman I. (2024). A signaling pathway map of plasminogen activator inhibitor-1 (PAI-1/SERPINE-1): A review of an innovative frontier in molecular aging and cellular senescence. Cell Commun. Signal..

[2] Sillen M., Declerck P.J. (2021). Targeting PAI-1 in Cardiovascular Disease: Structural Insights Into PAI-1 Functionality and Inhibition. Front. Cardiovasc. Med..

[3] Li S.-H., Reinke A.A., Sanders K.L., Emal C.D., Whisstock J.C., Stuckey J.A., Lawrence D.A. (2013). Mechanistic characterization and crystal structure of a small molecule inactivator bound to plasminogen activator inhibitor-1. Proc. Natl. Acad. Sci. USA.

[4] Loskutoff D.J., van Mourik J.A., Erickson L.A., Lawrence D. (1983). Detection of an unusually stable fibrinolytic inhibitor produced by bovine endothelial cells. Proc. Natl. Acad. Sci. USA.

[5] Andreasen P., Riccio A., Welinder K., Douglas R., Sartorio R., Nielsen L., Oppenheimer C., Blasi F., Danø K. (1986). Plasminogen activator inhibitor type-1: Reactive center and amino-terminal heterogeneity determined by protein and cDNA sequencing. FEBS Lett..

[6] Van De Craen B., Declerck P.J., Gils A. (2012). The Biochemistry, Physiology and Pathological roles of PAI-1 and the requirements for PAI-1 inhibition in vivo. Thromb. Res..

[7] Iwaki T., Urano T., Umemura K. (2012). PAI-1, progress in understanding the clinical problem and its aetiology. Br. J. Haematol..

[8] Vaughan D.E., Rai R., Khan S.S., Eren M., Ghosh A.K. (2017). Plasminogen Activator Inhibitor-1 Is a Marker and a Mediator of Senescence. Arter. Thromb. Vasc. Biol..

[9] Kohler H.P., Grant P.J. (2000). Plasminogen-activator inhibitor type 1 and coronary artery disease. N. Engl. J. Med..

[10] Bastard J.P., Piéroni L. (1999). Plasma plasminogen activator inhibitor 1, insulin resistance and android obesity. Biomed. Pharmacother..

[11] Alessi M.C., Peiretti F., Morange P., Henry M., Nalbone G., Juhan-Vague I. (1997). Production of plasminogen activator inhibitor 1 by human adipose tissue: Possible link between visceral fat accumulation and vascular disease. Diabetes.

[12] Robbie L.A., Bennett B., Croll A.M., Brown P.A.J., Booth N.A. (1996). Proteins of the fibrinolytic system in human thrombi. Thromb. Haemost..

[13] Helén B., Lena K., Maria A., Lingwei W., David E., Sverker J.J.B. (2004). Platelets synthesize large amounts of active plasminogen activator inhibitor 1. Blood.

[14] Sawdey M.S., Loskutoff D.J. (1991). Regulation of murine type 1 plasminogen activator inhibitor gene expression in vivo. Tissue specificity and induction by lipopolysaccharide, tumor necrosis factor-alpha, and transforming growth factor-beta. J. Clin. Investig..

[15] Booth N.A., Simpson A.J., Croll A., Bennett B., MacGregor I.R. (1988). Plasminogen activator inhibitor (PAI-1) in plasma and platelets. Br. J. Haematol..

[16] Li S., Wei X., He J., Tian X., Yuan S., Sun L. (2018). Plasminogen activator inhibitor-1 in cancer research. Biomed. Pharmacother..

[17] Jiang C.-S., Rana T., Jin L.-W., Farr S.A., Morley J.E., Qin H., Liu G., Liu R.-M. (2023). Aging, Plasminogen Activator Inhibitor 1, Brain Cell Senescence, and Alzheimer’s Disease. Aging Dis..

[18] Sillen M., Declerck P.J. (2021). A Narrative Review on Plasminogen Activator Inhibitor-1 and Its (Patho)Physiological Role: To Target or Not to Target?. Int. J. Mol. Sci..

[19] Chen X., Wang H., Wu C., Li X., Huang X., Ren Y., Pu Q., Cao Z., Tang X., Ding B.-S. (2024). Endothelial H(2)S-AMPK dysfunction upregulates the angiocrine factor PAI-1 and contributes to lung fibrosis. Redox Biol..

[20] Ma L.-J., Fogo A.B. (2009). PAI-1 and kidney fibrosis. Front. Biosci..

[21] Ibrahim A.A., Fujimura T., Uno T., Terada T., Hirano K.-I., Hosokawa H., Ohta A., Miyata T., Ando K., Yahata T. (2024). Plasminogen activator inhibitor-1 promotes immune evasion in tumors by facilitating the expression of programmed cell death-ligand 1. Front. Immunol..

[22] Okada K., Niwa Y., Fukuhara K., Ohira T., Mizukami Y., Kawao N., Matsuo O., Kaji H. (2024). Plasminogen activator inhibitor-1 is involved in glucocorticoid-induced decreases in angiogenesis during bone repair in mice. J. Bone Miner. Metab..

[23] Tamura Y., Kawao N., Okada K., Yano M., Okumoto K., Matsuo O., Kaji H. (2013). Plasminogen activator inhibitor-1 is involved in streptozotocin-induced bone loss in female mice. Diabetes.

[24] Hong G., Zhou Y., Yang S., Yan S., Lu J., Xu B., Zhan Z., Jiang H., Wei B., Wang J. (2024). Metformin acts on miR-181a-5p/PAI-1 axis in stem cells providing new strategies for improving age-related osteogenic differentiation decline. STEM CELLS.

[25] Campisi J. (2012). Aging, cellular senescence, and cancer. Annu. Rev. Physiol..

[26] Rana T., Jiang C., Liu G., Miyata T., Antony V., Thannickal V.J., Liu R.-M. (2020). PAI-1 Regulation of TGF-β1-induced Alveolar Type II Cell Senescence, SASP Secretion, and SASP-mediated Activation of Alveolar Macrophages. Am. J. Respir. Cell Mol. Biol..

[27] Zhang Q., Jin Y., Li X., Peng X., Peng N., Song J., Xu M. (2020). Plasminogen activator inhibitor-1 (PAI-1) 4G/5G promoter polymorphisms and risk of venous thromboembolism—A meta-analysis and systematic review. Vasa.

[28] Baglin T. (2012). Inherited and acquired risk factors for venous thromboembolism. Semin. Respir. Crit. Care Med..

[29] Wang Z., Kong L., Luo G., Zhang H., Sun F., Liang W., Wu W., Guo Z., Zhang R., Dou Y. (2022). Clinical impact of the PAI-1 4G/5G polymorphism in Chinese patients with venous thromboembolism. Thromb. J..

[30] Miri S., Sheikhha M.H., Dastgheib S.A., Shaker S.A., Neamatzadeh H. (2021). Association of ACE I/D and PAI-1 4G/5G polymorphisms with susceptibility to type 2 diabetes mellitus. J. Diabetes Metab. Disord..

[31] Ozgen M., Cosan D.T., Doganer F., Soyocak A., Armagan O., Gunes H.V., Degirmenci I., Ozkara G.O., Mutlu F.S. (2012). Relationship between plasminogen activator inhibitor type-1 (PAI-1) gene polymorphisms and osteoporosis in Turkish women. Clinics.

[32] Bae S.-C., Lee Y.H. (2019). Association between plasminogen activator inhibitor-1 (PAI-1) 4G/5G polymorphism and circulating PAI-1 level in systemic lupus erythematosus and rheumatoid arthritis: A meta-analysis. Z. Rheumatol..

[33] Mari D., Coppola R., Provenzano R. (2007). Hemostasis factors and aging. Exp. Gerontol..

[34] Lottermoser K., Düsing R., Ervens P., Koch B., Brüning T., Sachinidis A., Vetter H., Ko Y. (2001). The plasminogen activator inhibitor 1 4G/5G polymorphism is not associated with longevity: A study in octogenarians. J. Mol. Med..

[35] Nagai N., Suzuki Y., VAN Hoef B., Lijnen H.R., Collen D. (2005). Effects of plasminogen activator inhibitor-1 on ischemic brain injury in permanent and thrombotic middle cerebral artery occlusion models in mice. J. Thromb. Haemost..

[36] Dzhoyashvili N.A., Efimenko A.Y., Kochegura T.N., Kalinina N.I., Koptelova N.V., Sukhareva O.Y., Shestakova M.V., Akchurin R.S., Tkachuk V.A., Parfyonova Y.V. (2014). Disturbed angiogenic activity of adipose-derived stromal cells obtained from patients with coronary artery disease and diabetes mellitus type 2. J. Transl. Med..

[37] Rana T., Jiang C., Banerjee S., Yi N., Zmijewski J., Liu G., Liu R.J.C. (2023). PAI-1 Regulation of p53 Expression and Senescence in Type II Alveolar Epithelial Cells. Cells.

[38] Jiang C., Liu G., Luckhardt T., Antony V., Zhou Y., Carter A.B., Thannickal V.J., Liu R. (2017). Serpine 1 induces alveolar type II cell senescence through activating p53-p21-Rb pathway in fibrotic lung disease. Aging Cell.

[39] Huang W., Akhter H., Jiang C., MacEwen M., Ding Q., Antony V., Thannickal V., Liu R.J. (2015). Plasminogen activator inhibitor 1, fibroblast apoptosis resistance, and aging-related susceptibility to lung fibrosis. Exp. Gerontol..

[40] Jiang C., Liu G., Cai L., Deshane J., Antony V., Thannickal V., Liu R.J.T.A. (2021). j.o.p. Divergent Regulation of Alveolar Type 2 Cell and Fibroblast Apoptosis by Plasminogen Activator Inhibitor 1 in Lung Fibrosis. Am. J. Pathol..

[41] Cohen C., Le Goff O., Soysouvanh F., Vasseur F., Tanou M., Nguyen C., Amrouche L., Le Guen J., Saltel-Fulero O., Meunier T. (2021). Glomerular endothelial cell senescence drives age-related kidney disease through PAI-1. EMBO Mol. Med..

[42] Aobulikasimu A., Liu T., Piao J., Sato S., Ochi H., Okawa A., Tsuji K., Asou Y.J.S. (2023). r. SIRT6-PAI-1 axis is a promising therapeutic target in aging-related bone metabolic disruption. Sci. Rep..

[43] Chung Y.-P., Chen Y.-W., Weng T.-I., Yang R.-S., Liu S.-H. (2019). Arsenic induces human chondrocyte senescence and accelerates rat articular cartilage aging. Arch. Toxicol..

[44] Hirahata M., Osaki M., Kanda Y., Sugimoto Y., Yoshioka Y., Kosaka N., Takeshita F., Fujiwara T., Kawai A., Ito H. (2016). PAI-1, a target gene of miR-143, regulates invasion and metastasis by upregulating MMP-13 expression of human osteosarcoma. Cancer Med..

[45] Mashiko S., Kitatani K., Toyoshima M., Ichimura A., Dan T., Usui T., Ishibashi M., Shigeta S., Nagase S., Miyata T. (2015). Inhibition of plasminogen activator inhibitor-1 is a potential therapeutic strategy in ovarian cancer. Cancer Biol. Ther..

[46] Palmirotta R., Ferroni P., Savonarola A., Martini F., Ciatti F., Laudisi A., Sini V., Del Monte G., Guadagni F., Roselli M. (2009). Prognostic value of pre-surgical plasma PAI-1 (plasminogen activator inhibitor-1) levels in breast cancer. Thromb. Res..

[47] Rubina K.A., Sysoeva V.Y., Zagorujko E.I., Tsokolaeva Z.I., Kurdina M.I., Parfyonova Y.V., Tkachuk V.A. (2017). Increased expression of uPA, uPAR, and PAI-1 in psoriatic skin and in basal cell carcinomas. Arch. Dermatol. Res..

[48] Pavón M.A., Arroyo-Solera I., Téllez-Gabriel M., León X., Virós D., López M., Gallardo A., Céspedes M.V., Casanova I., López-Pousa A. (2015). Enhanced cell migration and apoptosis resistance may underlie the association between high SERPINE1 expression and poor outcome in head and neck carcinoma patients. Oncotarget.

[49] Zhang W., Xu J., Fang H., Tang L., Chen W., Sun Q., Zhang Q., Yang F., Sun Z., Cao L. (2017). Endothelial cells promote triple-negative breast cancer cell metastasis via PAI-1 and CCL5 signaling. FASEB J..

[50] Murano S., Thweatt R., Reis R.J.S., Jones R.A., Moerman E.J., Goldstein S. (1991). Diverse gene sequences are overexpressed in werner syndrome fibroblasts undergoing premature replicative senescence. Mol. Cell. Biol..

[51] Comi P., Chiaramonte R., Maier J.A. (1995). Senescence-dependent regulation of type 1 plasminogen activator inhibitor in human vascular endothelial cells. Exp. Cell Res..

[52] Xu D., Neville R., Finkel T. (2000). Homocysteine accelerates endothelial cell senescence. FEBS Lett..

[53] Sun T., Ghosh A.K., Eren M., Miyata T., Vaughan D.E. (2019). PAI-1 contributes to homocysteine-induced cellular senescence. Cell. Signal..

[54] McDonald A.P., Meier T.R., Hawley A.E., Thibert J.N., Farris D.M., Wrobleski S.K., Henke P.K., Wakefield T.W., Myers D.D. (2010). Aging is associated with impaired thrombus resolution in a mouse model of stasis induced thrombosis. Thromb. Res..

[55] Yanaka M., Honma T., Sato K., Shinohara N., Ito J., Tanaka Y., Tsuduki T., Ikeda I. (2011). Increased monocytic adhesion by senescence in human umbilical vein endothelial cells. Biosci. Biotechnol. Biochem..

[56] Ota H., Akishita M., Eto M., Iijima K., Kaneki M., Ouchi Y. (2007). Sirt1 modulates premature senescence-like phenotype in human endothelial cells. J. Mol. Cell. Cardiol..

[57] Chen H., Wan Y., Zhou S., Lu Y., Zhang Z., Zhang R., Chen F., Hao D., Zhao X., Guo Z. (2012). Endothelium-specific SIRT1 overexpression inhibits hyperglycemia-induced upregulation of vascular cell senescence. Sci. China Life Sci..

[58] Elzi D.J., Lai Y., Song M., Hakala K., Weintraub S.T., Shiio Y. (2012). Plasminogen activator inhibitor 1--insulin-like growth factor binding protein 3 cascade regulates stress-induced senescence. Proc. Natl. Acad. Sci. USA.

[59] Omer A., Patel D., Lian X.J., Sadek J., Di Marco S., Pause A., Gorospe M., Gallouzi I.E. (2018). Stress granules counteract senescence by sequestration of PAI-1. Embo Rep..

[60] Eren M., Boe A.E., Murphy S.B., Place A.T., Nagpal V., Morales-Nebreda L., Urich D., Quaggin S.E., Budinger G.R.S., Mutlu G.M. (2014). PAI-1-regulated extracellular proteolysis governs senescence and survival in Klotho mice. Proc. Natl. Acad. Sci. USA.

[61] Khan S., Shah S., Klyachko E., Baldridge A., Eren M., Place A., Aviv A., Puterman E., Lloyd-Jones D., Heiman M. (2017). SERPINE1A null mutation in protects against biological aging in humans. Sci. Adv..

[62] Vaughan D.E. (2005). PAI-1 and atherothrombosis. J. Thromb. Haemost..

[63] Khoukaz H.B., Vadali M., Schoenherr A., Ramirez-Perez F.I., Morales-Quinones M., Sun Z., Fujie S., Foote C.A., Lyu Z., Zeng S. (2024). PAI-1 Regulates the Cytoskeleton and Intrinsic Stiffness of Vascular Smooth Muscle Cells. Arter. Thromb. Vasc. Biol..

[64] Park M.Y., Herrmann S.M., Saad A., Eirin A., Tang H., Lerman A., Textor S.C., Lerman L.O. (2014). Biomarkers of kidney injury and klotho in patients with atherosclerotic renovascular disease. Clin. J. Am. Soc. Nephrol..

[65] Yu Y., Li W., Xu L., Wang Y. (2023). Circadian rhythm of plasminogen activator inhibitor-1 and cardiovascular complications in type 2 diabetes. Front. Endocrinol..

[66] Gifford C.C., Lian F., Tang J., Costello A., Goldschmeding R., Samarakoon R., Higgins P.J. (2021). PAI-1 induction during kidney injury promotes fibrotic epithelial dysfunction via deregulation of klotho, p53, and TGF-β1-receptor signaling. FASEB J..

[67] Liu R.-M. (2022). Aging, Cellular Senescence, and Alzheimer’s Disease. Int. J. Mol. Sci..

[68] Yanev P., Martin-Jimenez C., Vesga-Jimenez D.J., Zvinys L., Weinrich N., Cree M.A., Preuss T.M., Zhang X., Yepes M. (2024). Plasminogen activator inhibitor-1 mediates cerebral ischemia-induced astrocytic reactivity. Blood Flow Metab..

[69] Bi J., Cai W., Ma T., Deng A., Ma P., Han Y., Lou C., Wu L. (2019). Protective effect of vildagliptin on TNF-α-induced chondrocyte senescence. IUBMB Life.

[70] Inoue M., Sawada T., Uchima Y., Kimura K., Nishihara T., Tanaka H., Yashiro M., Yamada N., Ohira M., Hirakawa K. (2005). Plasminogen activator inhibitor-1 (PAI-1) gene transfection inhibits the liver metastasis of pancreatic cancer by preventing angiogenesis. Oncol. Rep..

[71] Bajou K., Maillard C., Jost M., Lijnen R.H., Gils A., Declerck P., Carmeliet P., Foidart J.-M., Noel A. (2004). Host-derived plasminogen activator inhibitor-1 (PAI-1) concentration is critical for in vivo tumoral angiogenesis and growth. Oncogene.

[72] Placencio V.R., DeClerck Y.A. (2015). Plasminogen Activator Inhibitor-1 in Cancer: Rationale and Insight for Future Therapeutic Testing. Cancer Res..

[73] López-Otín C., Blasco M.A., Partridge L., Serrano M., Kroemer G. (2013). The hallmarks of aging. Cell.

[74] Goldstein S., Moerman E.J., Fujii S., Sobel B.E. (1994). Overexpression of plasminogen activator inhibitor type-1 in senescent fibroblasts from normal subjects and those with Werner syndrome. J. Cell. Physiol..

[75] Abderrahmani R., François A., Buard V., Benderitter M., Sabourin J.-C., Crandall D.L., Milliat F. (2009). Effects of pharmacological inhibition and genetic deficiency of plasminogen activator inhibitor-1 in radiation-induced intestinal injury. Int. J. Radiat. Oncol..

[76] Elokdah H., Abou-Gharbia M., Hennan J.K., McFarlane G., Mugford C.P., Krishnamurthy G., Crandall D.L. (2004). Tiplaxtinin, a novel, orally efficacious inhibitor of plasminogen activator inhibitor-1: Design, synthesis, and preclinical characterization. J. Med. Chem..

[77] Ghosh A.K., Rai R., Park K.E., Eren M., Miyata T., Wilsbacher L.D., Vaughan D.E. (2016). A small molecule inhibitor of PAI-1 protects against doxorubicin-induced cellular senescence. Oncotarget.

[78] Daci E., Verstuyf A., Moermans K., Bouillon R., Carmeliet G. (2000). Mice lacking the plasminogen activator inhibitor 1 are protected from trabecular bone loss induced by estrogen deficiency. J. Bone Miner. Res..

[79] Mao L., Kawao N., Tamura Y., Okumoto K., Okada K., Yano M., Matsuo O., Kaji H. (2014). Plasminogen activator inhibitor-1 is involved in impaired bone repair associated with diabetes in female mice. PLoS ONE.

[80] Jin G., Aobulikasimu A., Piao J., Aibibula Z., Koga D., Sato S., Ochi H., Tsuji K., Nakabayashi T., Miyata T. (2018). A small-molecule PAI-1 inhibitor prevents bone loss by stimulating bone formation in a murine estrogen deficiency-induced osteoporosis model. FEBS Open Bio.

[81] Shimoide T., Kawao N., Tamura Y., Okada K., Horiuchi Y., Okumoto K., Kurashimo S., Ishida M., Tatsumi K., Matsuo O. (2018). Role of Macrophages and Plasminogen Activator Inhibitor-1 in Delayed Bone Repair in Diabetic Female Mice. Endocrinology.

[82] Ishiwata S., Iizuka H., Sonoda H., Tsunoda D., Tajika Y., Chikuda H., Koibuchi N., Shimokawa N. (2020). Upregulated miR-224-5p suppresses osteoblast differentiation by increasing the expression of Pai-1 in the lumbar spine of a rat model of congenital kyphoscoliosis. Mol. Cell. Biochem..

[83] Mizukami Y., Kawao N., Ohira T., Hashimoto D., Okada K., Matsuo O., Kaji H. (2024). Roles of Plasminogen Activator Inhibitor-1 in Heterotopic Ossification Induced by Achilles Tenotomy in Thermal Injured Mice. Calcif. Tissue Int..

[84] Nordstrom S., Carleton S., Carson W., Eren M., Phillips C., Vaughan D. (2007). Transgenic over-expression of plasminogen activator inhibitor-1 results in age-dependent and gender-specific increases in bone strength and mineralization. Bone.

[85] Takafuji Y., Tatsumi K., Ishida M., Kawao N., Okada K., Matsuo O., Kaji H. (2018). Plasminogen activator inhibitor-1 deficiency suppresses osteoblastic differentiation of mesenchymal stem cells in mice. J. Cell. Physiol..

[86] Li Z., Kegui H., Piao W., Xuejiu W., Lim K., Jin H. (2024). PAI-1 transfected-conditioned media promotes osteogenic differentiation of hBMSCs. Cell Biol. Int..

[87] Jin H., Xu Y., Qi Y., Wang X., Patel D.K., Dutta S.D., Chen R., Lim K.-T. (2020). Evaluation of Osteogenic/Cementogenic Modulating Potential of PAI-1 Transfected Media for Stem Cells. IEEE Trans. NanoBioscience.

[88] Njie R., Xu S., Wu T., Pi J., Lin S., Zhang P., Wang J., Dai Q., Shen H., Zhang N. (2025). Hedgehog Signalling in Osteogenesis and Bone Metabolism: Molecular Mechanisms, Regulatory Networks and Implications for Skeletal Disease. J. Cell. Mol. Med..

[89] Moon J.-S., Kim S.-H., Oh S.-H., Jeong Y.-W., Kang J.-H., Park J.-C., Son H.-J., Bae S., Park B.-I., Kim M.-S. (2014). Relaxin augments BMP-2-induced osteoblast differentiation and bone formation. J. Bone Miner. Res..

[90] Komori T. (2010). Regulation of bone development and extracellular matrix protein genes by RUNX2. Cell Tissue Res..

[91] Moritake A., Kawao N., Okada K., Tatsumi K., Ishida M., Okumoto K., Matsuo O., Akagi M., Kaji H. (2017). Plasminogen activator inhibitor-1 deficiency enhances subchondral osteopenia after induction of osteoarthritis in mice. BMC Musculoskelet. Disord..

[92] Hadadeh O., Barruet E., Peiretti F., Verdier M., Bernot D., Hadjal Y., El Yazidi C., Robaglia-Schlupp A., De Paula A.M., Nègre D. (2012). The plasminogen activation system modulates differently adipogenesis and myogenesis of embryonic stem cells. PLoS ONE.

[93] Kusuyama J., Bandow K., Ohnishi T., Amir M.S., Shima K., Semba I., Matsuguchi T. (2019). CXCL13 is a differentiation- and hypoxia-induced adipocytokine that exacerbates the inflammatory phenotype of adipocytes through PHLPP1 induction. Biochem. J..

[94] Gallego-Escuredo J.M., Gutierrez M.d.M., Diaz-Delfin J., Domingo J.C., Mateo M.G., Domingo P., Giralt M., Villarroya F. (2010). Differential effects of efavirenz and lopinavir/ritonavir on human adipocyte differentiation, gene expression and release of adipokines and pro-inflammatory cytokines. Curr. HIV Res..

[95] Zhra M., Magableh A.M., Samhan L.M., Fatani L.M., Qasem R.J., Aljada A. (2024). The Expression of a Subset of Aging and Antiaging Markers Following the Chondrogenic and Osteogenic Differentiation of Mesenchymal Stem Cells of Placental Origin. Cells.

[96] Lee S.H., Ko H.M., Kwon K.J., Lee J., Han S.-H., Han D.W., Cheong J.H., Ryu J.H., Shin C.Y. (2014). tPA regulates neurite outgrowth by phosphorylation of LRP5/6 in neural progenitor cells. Mol. Neurobiol..

[97] Ko H.M., Joo S.H., Lee S.H., Kim H.J., Lee S.-H., Cheong J.H., Ryu J.H., Kim J.M., Koo B.-N., Shin C.Y. (2015). Propofol treatment modulates neurite extension regulated by immunologically challenged rat primary astrocytes: A possible role of PAI-1. Arch. Pharmacal Res..

[98] Koyanagi S., Kuramoto Y., Kimura M., Oda M., Kozako T., Hayashida S., Shimeno H., Soeda S. (2017). Anti-apoptotic roles of plasminogen activator inhibitor-1 as a neurotrophic factor in the central nervous system. Thromb. Haemost..

[99] Genestine M., Ambriz D., Crabtree G.W., Dummer P., Molotkova A., Quintero M., Mela A., Biswas S., Feng H., Zhang C. (2021). Vascular-derived SPARC and SerpinE1 regulate interneuron tangential migration and accelerate functional maturation of human stem cell-derived interneurons. eLife.

[100] Niaz A., Karunia J., Mandwie M., Keay K.A., Musumeci G., Al-Badri G., Castorina A. (2021). Robust Dopaminergic Differentiation and Enhanced LPS-Induced Neuroinflammatory Response in Serum-Deprived Human SH-SY5Y Cells: Implication for Parkinson’s Disease. J. Mol. Neurosci..

[101] Adibhatla R.M., Hatcher J.F. (2008). Tissue plasminogen activator (tPA) and matrix metalloproteinases in the pathogenesis of stroke: Therapeutic strategies. CNS Neurol. Disord.—Drug Targets.

[102] Achuta V.S., Rezov V., Uutela M., Louhivuori V., Louhivuori L., Castrén M.L. (2014). Tissue Plasminogen Activator Contributes to Alterations of Neuronal Migration and Activity-Dependent Responses in Fragile X Mice. J. Neurosci..

[103] Lemaire R., Burwell T., Sun H., Delaney T., Bakken J., Cheng L., Rebelatto M.C., Czapiga M., De-Mendez I., Coyle A.J. (2015). Resolution of Skin Fibrosis by Neutralization of the Antifibrinolytic Function of Plasminogen Activator Inhibitor 1. Arthritis Rheumatol..

[104] Sun Y., Wang Y., Long J., Wang X. (2014). Association of the plasminogen activator inhibitor-1 (PAI-1) Gene -675 4G/5G and -844 A/G promoter polymorphism with risk of keloid in a Chinese Han population. Med Sci. Monit..

[105] Liu R.-M. (2007). Oxidative stress, plasminogen activator inhibitor 1, and lung fibrosis. Antioxidants Redox Signal..

[106] Courey A.J., Horowitz J.C., Kim K.K., Koh T.J., Novak M.L., Subbotina N., Warnock M., Xue B., Cunningham A.K., Lin Y. (2011). The vitronectin-binding function of PAI-1 exacerbates lung fibrosis in mice. Blood.

[107] Małgorzewicz S., Skrzypczak-Jankun E., Jankun J. (2013). Plasminogen activator inhibitor-1 in kidney pathology (Review). Int. J. Mol. Med..

[108] Bergheim I., Guo L., Davis M.A., Duveau I., Arteel G.E. (2005). Critical role of plasminogen activator inhibitor-1 in cholestatic liver injury and fibrosis. J. Pharmacol. Exp. Ther..

[109] Bauman K.A., Wettlaufer S.H., Okunishi K., Vannella K.M., Stoolman J.S., Huang S.K., Courey A.J., White E.S., Hogaboam C.M., Simon R.H. (2010). The antifibrotic effects of plasminogen activation occur via prostaglandin E2 synthesis in humans and mice. J. Clin. Investig..

[110] Takeshita K., Hayashi M., Iino S., Kondo T., Inden Y., Iwase M., Kojima T., Hirai M., Ito M., Loskutoff D.J. (2004). Increased expression of plasminogen activator inhibitor-1 in cardiomyocytes contributes to cardiac fibrosis after myocardial infarction. Am. J. Pathol..

[111] Ghosh A.K., Bradham W.S., Gleaves L.A., De Taeye B., Murphy S.B., Covington J.W., Vaughan D.E. (2010). Genetic deficiency of plasminogen activator inhibitor-1 promotes cardiac fibrosis in aged mice: Involvement of constitutive transforming growth factor-beta signaling and endothelial-to-mesenchymal transition. Circulation.

[112] Han J.-Y., Kim Y.-J., Kim L., Choi S.-J., Park I.-S., Kim J.-M., Chu Y.C., Cha D.-R. (2010). PPARgamma agonist and angiotensin II receptor antagonist ameliorate renal tubulointerstitial fibrosis. J. Korean Med Sci..

[113] Zhang G., Cheng T., Luan Q., Liao T., Nie C., Zheng X., Xie X., Gao W. (2010). Vitamin D: A novel therapeutic approach for keloid, an in vitro analysis. Br. J. Dermatol..

[114] Kortlever R.M., Higgins P.J., Bernards R. (2006). Plasminogen activator inhibitor-1 is a critical downstream target of p53 in the induction of replicative senescence. Nat. Cell Biol..

[115] Liu F., Wu S., Ren H., Gu J. (2011). Klotho suppresses RIG-I-mediated senescence-associated inflammation. Nat. Cell Biol..

[116] López-Andrés N., Calvier L., Labat C., Fay R., Díez J., Benetos A., Zannad F., Lacolley P., Rossignol P. (2012). Absence of cardiotrophin 1 is associated with decreased age-dependent arterial stiffness and increased longevity in mice. Hypertension.

[117] Born E., Lipskaia L., Breau M., Houssaini A., Beaulieu D., Marcos E., Pierre R., Cruzeiro M.D., Lefevre M., Derumeaux G. (2023). Eliminating Senescent Cells Can Promote Pulmonary Hypertension Development and Progression. Circulation.

[118] Eitzman D.T., McCoy R.D., Zheng X., Fay W.P., Shen T., Ginsburg D., Simon R.H. (1996). Bleomycin-induced pulmonary fibrosis in transgenic mice that either lack or overexpress the murine plasminogen activator inhibitor-1 gene. J. Clin. Investig..

[119] Oda T., Jung Y.O., Kim H.S., Cai X., López-Guisa J.M., Ikeda Y., Eddy A.A. (2001). PAI-1 deficiency attenuates the fibrogenic response to ureteral obstruction. Kidney Int..

[120] Eddy A.A., Fogo A.B. (2006). Plasminogen activator inhibitor-1 in chronic kidney disease: Evidence and mechanisms of action. J. Am. Soc. Nephrol..

[121] Hu J., Liu Y., Pan Z., Huang X., Wang J., Cao W., Chen Z. (2023). Eupatilin Ameliorates Hepatic Fibrosis and Hepatic Stellate Cell Activation by Suppressing β-catenin/PAI-1 Pathway. Int. J. Mol. Sci..

[122] Xu Z., Castellino F.J., Ploplis V.A. (2009). Plasminogen activator inhibitor-1 (PAI-1) is cardioprotective in mice by maintaining microvascular integrity and cardiac architecture. Blood.

[123] Pedroja B.S., Kang L.E., Imas A.O., Carmeliet P., Bernstein A.M. (2009). Plasminogen activator inhibitor-1 regulates integrin alphavbeta3 expression and autocrine transforming growth factor beta signaling. J. Biol. Chem..

[124] Yepes M., Roussel B.D., Ali C., Vivien D. (2009). Tissue-type plasminogen activator in the ischemic brain: More than a thrombolytic. Trends Neurosci..

[125] Khoddam A., Miyata T., Vaughan D. (2025). PAI-1 is a common driver of aging and diverse diseases. Biomed. J..

[126] Rao J.S., Rayford A., Morantz R.A., Festoff B.W., Sawaya R. (1993). Increased levels of plasminogen activator inhibitor-1 (PAI-1) in human brain tumors. J. Neuro-Oncol.

[127] Marie P.J. (2008). Transcription factors controlling osteoblastogenesis. Arch. Biochem. Biophys..

